# Microgravity environment grown crystal structure information based engineering of direct electron transfer type glucose dehydrogenase

**DOI:** 10.1038/s42003-022-04286-9

**Published:** 2022-12-06

**Authors:** Junko Okuda-Shimazaki, Hiromi Yoshida, Inyoung Lee, Katsuhiro Kojima, Nanoha Suzuki, Wakako Tsugawa, Mitsugu Yamada, Koji Inaka, Hiroaki Tanaka, Koji Sode

**Affiliations:** 1 Joint Department of Biomedical Engineering, The University of North Carolina at Chapel Hill and North Carolina State University, Chapel Hill, NC27599 USA; 2 Department of Basic Life Science, Faculty of Medicine, Kagawa University, 1750-1 Ikenobe, Miki-cho, Kita-gun, Kagawa 761-0793 Japan; 3 Graduate School of Engineering, Department of Biotechnology and Life Science, Tokyo University of Agriculture and Technology, Koganei, Tokyo 184-8588 Japan; 4 JEM Utilization Center Human Spaceflight Technology Directorate, Japan Aerospace Exploration Agency (JAXA), 2-1-1 Sengen, Tsukuba-shi, Ibaraki 305-8505 Japan; 5 Maruwa Foods and Biosciences, 170-1 Tsutsui-cho, Yamato Koriyama-shi, Nara 639-1123 Japan; 6 Confocal Science Inc., Musashino Bldg, 5-14-15 Fukasawa, Setagaya-ku, Tokyo 158-0081 Japan

**Keywords:** Protein design, Oxidoreductases

## Abstract

The heterotrimeric flavin adenine dinucleotide dependent glucose dehydrogenase is a promising enzyme for direct electron transfer (DET) principle-based glucose sensors within continuous glucose monitoring systems. We elucidate the structure of the subunit interface of this enzyme by preparing heterotrimer complex protein crystals grown under a space microgravity environment. Based on the proposed structure, we introduce inter-subunit disulfide bonds between the small and electron transfer subunits (5 pairs), as well as the catalytic and the electron transfer subunits (9 pairs). Without compromising the enzyme’s catalytic efficiency, a mutant enzyme harboring Pro205Cys in the catalytic subunit, Asp383Cys and Tyr349Cys in the electron transfer subunit, and Lys155Cys in the small subunit, is determined to be the most stable of the variants. The developed engineered enzyme demonstrate a higher catalytic activity and DET ability than the wild type. This mutant retains its full activity below 70 °C as well as after incubation at 75 °C for 15 min – much higher temperatures than the current gold standard enzyme, glucose oxidase, is capable of withstanding.

## Abstract (web summary)

Protein crystal growth experiments in a microgravity environment provide insight into the design of an engineered direct electron transfer type glucose dehydrogenase with increase thermal stability for glucose sensor applications.

## Introduction

Technological developments with respect to electrochemical glucose sensors are largely dependent on the continued innovation of the capabilities of glucose-sensing enzymes^1^. Three different electrochemical sensing principles for enzymatic glucose sensors have been reported: first-generation sensors utilize oxygen as an electron acceptor; second-generation sensors employ artificial electron acceptors or mediators; and third-generation sensors use neither oxygen nor mediators, but rather electrons can transfer directly to the electrode surface from the enzyme. Glucose sensors that are currently on the commercial market for diabetes management, such as disposable sensor strips for self-monitoring of blood glucose (SMBG), and implantable sensors for continuous glucose monitoring (CGM) systems, employ first- and second-generation principles^1,2^. Generally, these electrochemical sensors are developed by utilizing glucose oxidase (GOx) (β-D-glucose:oxygen 1-oxidoreductase, EC 1.1.3.4)^3^ or various glucose dehydrogenases (GDHs) which possess either nicotine adenine dinucleotide (NAD), nicotine adenine dinucleotide phosphate (NADP), pyrroloquinoline quinone (PQQ) or flavin adenine dinucleotide (FAD) as their cognate redox cofactors. GDHs harboring FAD (FADGDH) as their redox cofactor can be divided into three distinct groups according to their origin: bacterial, fungal, and insect. Considering the substrate specificity and availability of various electron mediators, fungi-derived FADGDHs are currently the most popular enzymes for use in sensor strips in second-generation SMBG sensors. Commercially available electrochemical implantable sensors for CGM systems rely on first- or second-generation principles, and they all utilize GOx, mainly due to its inherent stability^2^.

Third generation glucose sensors, which employ a glucose oxidizing enzyme capable of direct electron transfer (DET) with the electrode, are ideal for CGM systems compared to first- and second-generation sensors because of several key advantages^4^. However, the availability of glucose oxidoreductases inherently capable of DET is limited. One of the most prominent GDHs capable of DET is the bacteria-derived FADGDH complex, with the first reported example derived from *Burkholderia cepacia* (BcGDH)^4–14^. BcGDH is a glucose oxidoreductase with dye-mediated dehydrogenase activity, but lacks oxidase activity since it is unable to utilize oxygen as an electrode acceptor. The DET ability of this enzyme was first demonstrated by immobilizing the enzyme with carbon paste to an electrode in the absence of any additional electron acceptor, and the catalytic current responded in a glucose concentration-dependent manner^13^. Since then, various DET-type biodevices have been reported using BcGDH, such as enzyme fuel cell-based sensing^8^, BioCapacitor^9,10^, amperometirc sensing^11^, open circuit potential based sensing^12^, and impedimetric sensing^15^. Bacteria-derived FADGDH complexes are composed of three distinct subunits: a catalytic subunit harboring an FAD cofactor in its catalytic center with an iron sulfur cluster (Fe-S; 3Fe-4S) as the primary electron acceptor of FAD, a small subunit which is a hitch-hiker protein necessary for the bacterial TAT secretion system, and an electron transfer subunit harboring three heme *c* units. This enzyme can transfer electrons liberated from glucose oxidation directly to the electrode without any redox mediator as a consequence of the architecture of the electron transfer subunit. The electron transfer subunit is required for DET, and followingly, the dissociation of the electron transfer subunit eliminates BcGDH’s DET ability. For BcGDH to be suitable for CGM systems, destabilization of the quaternary structure of BcGDH, specifically the dissociation of the electron transfer subunit from the catalytic subunit conjugated with the small subunit through a disulfide bond, should be prevented.

We recently reported the crystal structure of the BcGDH catalytic and small subunit complex (PDB:6a2u)^16^. Through structural analysis, an intrinsic disulfide bond between the catalytic (Cys213) and small subunit (Cys152) was determined. The cysteine residue forming the disulfide bond in the catalytic subunit is located in proximity to the cysteine residues which form the Fe-S cluster. In the structure of the catalytic/small subunit complex, the Fe-S cluster and this disulfide bond are located at the surface of the catalytic subunit. Mutagenesis studies revealed that the cysteine residue which forms the disulfide bond between catalytic and small subunits is critical for stability at high temperatures, whereas catalytic activity of the mutant was comparable to the wild type at room temperature^17^. From this observation, we concluded that the intrinsic disulfide bond between the catalytic and small subunits in BcGDH might stabilize the catalytic subunit, especially its Fe-S cluster.

We also reported previously that chemical crosslinking of BcGDH with glutaraldehyde improved its thermal stability^18^. This observation suggested that quaternary structure stabilization may be achieved via the construction of a stable BcGDH complex harboring an electron transfer subunit. However, the rate of chemical crosslinking processes is difficult to control and random modification of residues can occur, which could result in a decrease in catalytic activity or change in DET ability. A more reliable method to stabilize the quaternary structure of this enzyme would be to introduce disulfide bonds among the residues of its inter-subunit regions: either between the catalytic and electron transfer subunits and/or the small and electron transfer subunits. We hypothesize that these disulfide bonds—in addition to the intrinsic disulfide bond between the catalytic and small subunits—will improve enzyme stability without compromising its catalytic activity or DET ability. However, the crystal structure of a BcGDH complex with an electron transfer subunit, or any homologous FAD-dependent dehydrogenase complex harboring multiple heme *c* electron transfer subunits, has yet to be elucidated, making it a challenge to design such disulfide bonds. The nature of these enzymes proves crystal formation difficult: FAD-dependent dehydrogenase complexes are membrane-bound hydrophobic enzymes and also form heterogeneous complexes in solution.

The Japan Aerospace Exploration Agency (JAXA) has been conducting High-quality Protein Crystal Growth experiments (JAXA PCG) in the Japanese Kibo module on the International Space Station (ISS). Owing to the unique environment in space where there is no convection to disrupt liquid solution nor precipitation causing heavier molecules to sink, many crystals of various proteins have been successfully created in this way^19–24^. To improve the stability of the DET-type FADGDH complex via quaternary structure engineering, we elucidated the partial structure of this enzyme complex by preparing a protein crystal of BcGDH heterotrimer complex in space through JAXA PCG experiments. Utilizing the provisional crystal structure containing a partial structure of the third heme *c*, within the electron transfer subunit, which is the primary electron acceptor of the iron sulfur cluster in the catalytic subunit, we constructed a structural model showing the putative inter-subunit region. Based on the structural model, 14 candidate amino acid residues were selected to form disulfide bond pairs between the small and electron transfer subunits (5 pairs), as well as the catalytic and electron transfer subunits (9 pairs).

The engineering of BcGDH resulted in enzyme super-stabilization: the constructed mutant did not lose activity for at least 30 minutes in solution at 70 °C, making its thermal stability much higher than GOx. Surprisingly, the mutant enzyme showed 70% higher catalytic efficiency compared with the control BcGDH. We conjecture that this phenomenon is attributed to the disulfide bond formation which shortens the distance between the catalytic subunit and electron transfer subunit, and also possible contribution of disulfide bond to foster inter-molecular electron transfer. Electrochemical evaluation of enzyme-functionalized electrodes with the mutant BcGDH confirmed its DET ability, with a higher current density than those electrodes with control BcGDH alone. This DET-type FADGDH with engineered quaternary structure, based on the structural information obtained from crystals grown in a space microgravity environment, is a promising enzyme candidate for future third-generation CGM systems.

## Results

### Tertiary and quaternary structural analyses of BcGDH complex composed of γα and partial β/design of disulfide bond

**Fig. 1:**
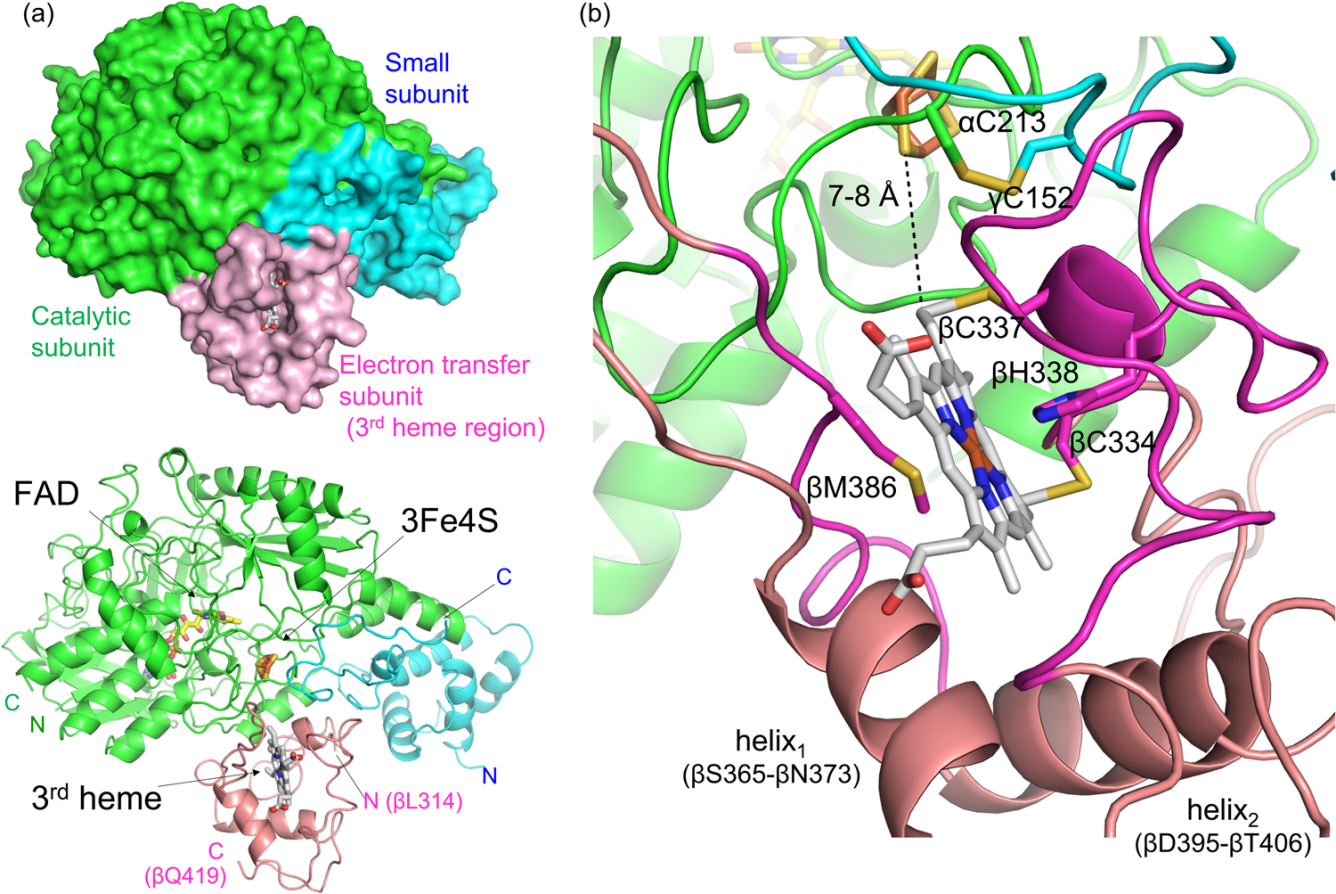
Structure model of BcGDH heterotrimer complex. **a** Overall structure of the constructed model based on crystal structure analysis. Surface model (up) and cartoon model (down) are shown. **b** Modeled structure around partial electron transfer subunit. αCys213 and γCys152 form an intrinsic disulfide bond between catalytic and small subunits as previously reported. βCys334 and βCys337 likely interact with the thioether of the third heme in the catalytic subunit. βHis338 and βMet386 are predicted as the two axial ligands of the third heme iron. α denotes the catalytic subunit, γ denotes the small subunit, and β denotes the electron transfer subunit; colored with green, cyan, and pink, respectively. FAD and 3Fe4S are shown in yellow stick model, third heme *c* is shown in white stick model. N and C indicate N-terminus and C-terminus of each subunit, respectively. In the electron transfer subunit, helix_1_ (βSer365-βAsn373), helix_2_ (βAsp395-βThr406), and two regions colored with magenta (βCys334 to βThr358 and βIle384 to βAsn394) surround the third heme *c*.

Figure 1 shows the proposed structure of a BcGDH complex composed of the catalytic, small, and third heme *c* region of the electron transfer subunit; hereafter, α denotes the catalytic subunit, γ denotes the small subunit, and β denotes the electron transfer subunit. The proposed structure is modeled based on X-ray diffraction data analyses of the BcGDH complex crystal grown using the crystallization facility of JAXA on board the Japanese Experiment Module Kibo on the ISS. Through electron density analysis, a partial structure of the electron transfer subunit from βLeu314 to the C-terminal end colored in pink (Fig. 1a) was determined, containing the third heme *c*. Two helices (helix_1_ and helix_2_) and two regions colored with magenta (βCys334 to βThr358 and βIle384 to βAsn394) surround the third heme *c* (Fig. 1b). βHis338 and βMet386 are predicted as the two axial ligands of the heme iron, and each ligand is located at the above mentioned two colored-regions with magenta. Heme is covalently bound to the enzyme via two cysteine residues (βCys334 and βCys337), which is typical of heme binding motifs, CXXCH^25,26^. The overall structure revealed the third heme *c* region of the electron transfer subunit interacts with both catalytic and small subunits (Fig. 1). The thioether end of the third heme *c* is oriented to the Fe-S cluster, and the region from βCys334 to βThr358, colored with magenta including the heme binding motif, interacts with the small subunit colored with cyan. This region is close to the intrinsic disulfide bond formed between αC213 and γC152. The catalytic subunit colored with green mainly interacts with the region between helix_1_ and helix_2_ and includes the colored-region in magenta (βIle384 to βAsn394), where the sixth ligand (βM386) of the third heme is located. From the overall complex structure, the location of each cofactor and their distances were estimated. The edge-to-edge distance between the third heme *c* and the 3Fe-4S in the catalytic subunit is 7~8 Å. Because of this short distance, it is expected electrons are directly transferred between the 3Fe-4S of the catalytic subunit to the third heme *c* in the electron transfer subunit.

**Fig. 2:**
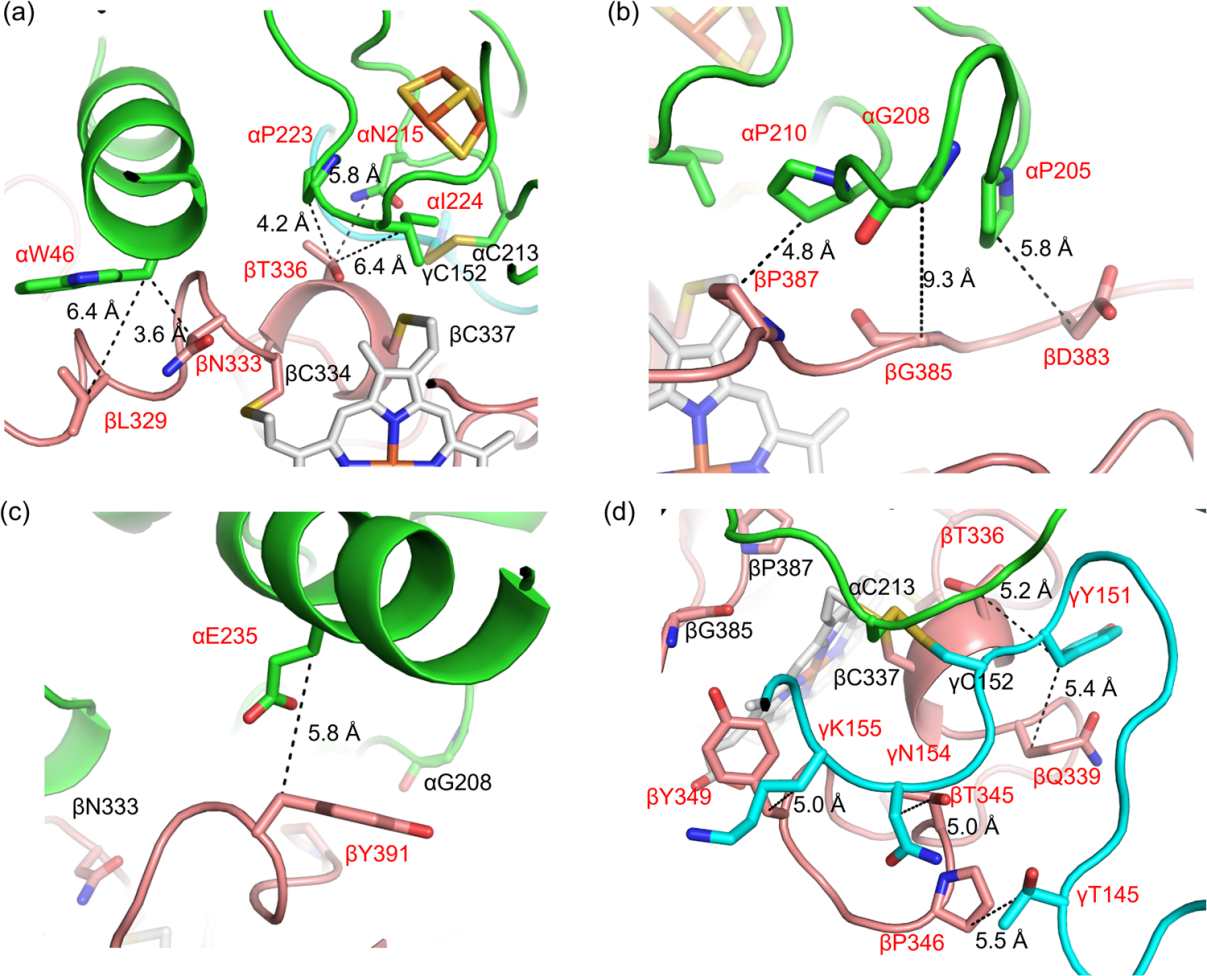
Designed disulfide bonds in BcGDHγαβ complex. Dotted lines show the distances of around 5 Å between C_β_ of selected residues with red labels. The colors of cartoon and stick models are the same as presented in Fig. 1a. 9 pairs between catalytic and electron transfer subunits are described in (**a**) to (**c**); **a** αW46/βL329 (6.4 Å), αW46/βN333 (3.6 Å), αN215/βT336 (5.8 Å), αP223/βT336 (4.2 Å), αI224/βT336 (6.4 Å), **b** αP205/βD383 (5.8 Å), αG208/βG385 (C_α_s; 9.3 Å), αP210/βP387 (4.8 Å), **c** αE235/βY391 (5.8 Å), 5 pairs between small and electron transfer subunits are described in (**d**); **d** γT145/βP346 (5.5 Å), γY151/βT336 (5.2 Å), γY151/βQ339 (5.4 Å), γN154/βT345 (5.0 Å), γK155/Y349 (5.0 Å).

Based on the proposed tertiary and quaternary structure, we designed additional disulfide bonds between catalytic and electron transfer subunits, and small and electron transfer subunits (Fig. 2a–d). To find the residue pairs which would spontaneously form disulfide bonds when they are substituted to cysteine residues, we searched for a combination of residues which exist in proximity of around 5 Å between β-carbons (C_β_s) considering that the length of typical disulfide bonds is <5 Å^27,28^. We focused on the distance between C_β_s for the proximity search considering the orientation of each residues’ side chain. Our strategy was based on the following criteria: (1) choosing residue pairs existing in each subunit interface, (2) selecting residue pairs whose distance to each C_β_ is around 5 Å, (3) if the pair contains glycine, then selecting residue pairs whose distance between C_α_/C_α_ is <10 Å. In general, the average distance of disulfide bonds between C_β_/C_β_ pairs is about 3.5–4.5 Å, however, a clear indication of the distance of C_β_/C_β_ pairs before disulfide bond formation has not been reported. Therefore, in this study, we provisionally hypothesized that C_β_/C_β_ pairs within about 5 Å may form disulfide bonds when substituted to cystine residues.

To facilitate electron transfer from the 3Fe-4S to the third heme *c* while considering the limited surface area between small and electron transfer subunits, we selected the following nine pairs between catalytic and electron transfer subunits: αTrp46/βLeu329 (6.4 Å), αTrp46/βAsn333 (3.6 Å), αPro205/βAsp383 (5.8 Å), αGly208/βGly385 (C_α_s; 9.3 Å), αPro210/βPro387 (4.8 Å), αAsn215/βThr336 (5.8 Å), αPro223/βThr336 (4.2 Å), αIle224/βThr336 (6.4 Å), αGlu235/βTyr391 (5.8 Å), and selected five pairs between small and electron transfer subunits: γThr145/βPro346 (5.5 Å), γTyr151/βThr336 (5.2 Å), γTyr151/βGln339 (5.4 Å), γAsn154/βThr345 (5.0 Å), γLys155/βTyr349 (5.0 Å) (Supplementary Table 1). We then substituted these selected residues to Cys residues and recombinantly prepared 14 mutant BcGDH complexes and investigated their catalytic activities and thermal stabilities.

### Evaluation of designed mutants with disulfide bonds between α/β or γ/β subunits

We evaluated enzyme activity and thermal stability of designed mutants with cysteine-substituted pairs (Table 1) via recombinantly prepared crude enzyme samples expressed in *E.coli* (Supplementary Fig. 1a; uncropped and unedited gel images are shown in Supplementary Fig. 1b). Among constructed mutants, 50% of mutants (αW46C/βL329C, αP210C/βP387C, αN215C/βT336C, αP223C/βT336C, αI224C/βT336C, γT151C/βT336C, γY151C/βQ339C), showed <10% activity of parental BcGDH complex formation, indicating that these mutations resulted in inactivation or lack of proper soluble protein folding. Among the mutants with a designed disulfide bond between catalytic/electron transfer subunits, αP205C/βD383C showed remarkable improvement in thermal stability. This mutant demonstrated the highest residual activity, retaining more than 70% of its initial activity after heat treatment at 65 °C for 15 min (Table 1). αG208C/βG385C and αE235C/βY391C also showed improved thermal stability (>25%). Notably, βD383, βG385 and βY391 are in the region colored with magenta (Fig. 1b). We postulate the stabilizing interaction between this region and the catalytic subunit largely contributes to the stability of the overall quaternary structure. Interestingly, the small/electron transfer subunit disulfide bond mutants also resulted in thermostable improvement. γK155C/βY349C showed the highest stability (>80%) among small/electron transfer subunit mutants. γT145C/βP346C and γN154C/βT345C also showed improved thermal stability (>25%).

**Table 1:** Residual activity after treatment at 65 °C for 15 min.

Variants	Activity at 25 °C (U mg^−1^)	Residual activity (%)
γαβ control	9.08	1.7
αW46C/βL329C	0.23	4.4
αW46C/βN333C	4.47	7.2
αP205C/βD383C	8.14	70.4
αG208C/βG385C	5.62	24.6
αP210C/βP387C	0.44	2.1
αN215C/βT336C	0.87	9.9
αP223C/βT336C	0.81	1.2
αI224C/βT336C	0.62	1.6
αE235C/βY391C	8.92	36.8
γT145C/βP346C	7.96	25.9
γY151C/βT336C	0.02	36.9
γY151C/βQ339C	0.93	0.8
γN154C/βT345C	3.88	26.5
γK155C/βY349C	5.50	79.6
αP205C/βD383C- γK155C/βY349C	2.66	162.2

Crude enzyme samples were prepared by small-scale culture (3 mL). Enzyme activity was measured using Ru/MTT as the electron acceptors at 25 °C. Protein concentration of prepared soluble fraction was adjusted to 0.45 mg/mL for heat treatment at 65 ˚C (*n* = 3, 3 different clones).

To investigate the impact of protein sample concentration on the thermal stability of each mutant, the overall protein concentration of soluble fractions containing mutant enzymes were analyzed by SDS-PAGE (Supplementary Fig. 1a; uncropped and unedited gel images are shown in Supplementary Fig. 1b). We calculated the relative intensity of the bands corresponding to the catalytic subunit of each mutant compared to the control enzyme sample, which is denoted as 100% intensity (Supplementary Table 2). These results demonstrate that the expression levels of control enzyme (lane 1), γT145C/βP346C (lane 6), αW46C/βL329C (lane 8), αN215C/βT336C (lane 11) and γY151C/βT336C (lane 14) were higher than other mutant enzymes, including αP205C/βD383C (lane 3), and γK155C/βY349C (lane 4), which we selected as the stable mutants. Therefore, we observed increased stability of the selected mutants with less protein concentration than the control enzymes.

These results indicate that most of the designed disulfide bonds based on the predicted model of BcGDH correctly formed and contributed to constructing an engineered BcGDH with increased thermal stability. In addition, these results support the accuracy of the predicted partial quaternary structure of BcGDH.

We then explored the synergistic effect of both small/electron transfer subunit and catalytic/electron transfer subunit disulfide bond formations on thermal stability. We constructed a multi-mutant complex in which we combined the disulfide bonds designed between small/electron transfer subunits and catalytic/electron transfer subunits, by combining αP205C/βD383C and γK155C/βY349C. The crude enzyme sample of the designed mutant with two additional disulfide bonds showed the highest thermal stability of the constructed mutants in this study. However, the enzyme activity of αP205C/βD383C-γK155C/βY349C shown in Table 1 is 2.66 U  mg^−1^, whereas the control enzyme activity is 9.08 U mg^−1^; the mutant enzyme demonstrates only 30% activity of the control enzyme. However, as these values were obtained from measurement of crude enzyme samples, these values were affected by the expression level of each preparation. The expression level of the control enzyme is much higher than the mutant αP205C/βD383C-γK155C/βY349C (Supplementary Fig. 1a; uncropped and unedited gel images are shown in Supplementary Fig. 1b). Therefore, the lower catalytic activity observed is due to the relatively low expression level of the mutant enzyme in this sample preparation. The enzyme was not inactivated during incubation at 65 °C for 15 min, but it increased (162%), likely due to the appropriate conformational formation during thermal treatment. We then subjected the combined mutant, quaternary structure engineered BcGDH, further kinetic and electrochemical investigations.

### Characterization of quaternary structure engineered BcGDH

The purified quaternary structure engineered BcGDH (Supplementary Fig. 2a; uncropped and unedited gel images are shown in Supplementary Fig. 2b, Supplementary Table 3) exhibited a *k*_cat_ value of 602 s^−1^, with a catalytic efficiency of 42.4 s^−1 ^mM^−1^, both of which are higher than the BcGDH complex without additional disulfide bonds (*k*_cat_ = 237 s^−1^, *k*_cat_/Km = 24.3 s^−1 ^mM^−1^) (Table 2, Supplementary Fig. 3). These results suggest the two additional disulfide bonds did not negatively impact the kinetic parameters of BcGDH, but rather increased its catalytic efficiency.

**Table 2:** Kinetic parameters (at 25 °C).

	Km (mM)	*k*_cat_ (s^−1^)	*k*_cat_/Km
γK155C/βY349C αP205C/βD383C	14.2	602	42.4
Control	9.75	237	24.3

**Fig. 3:**
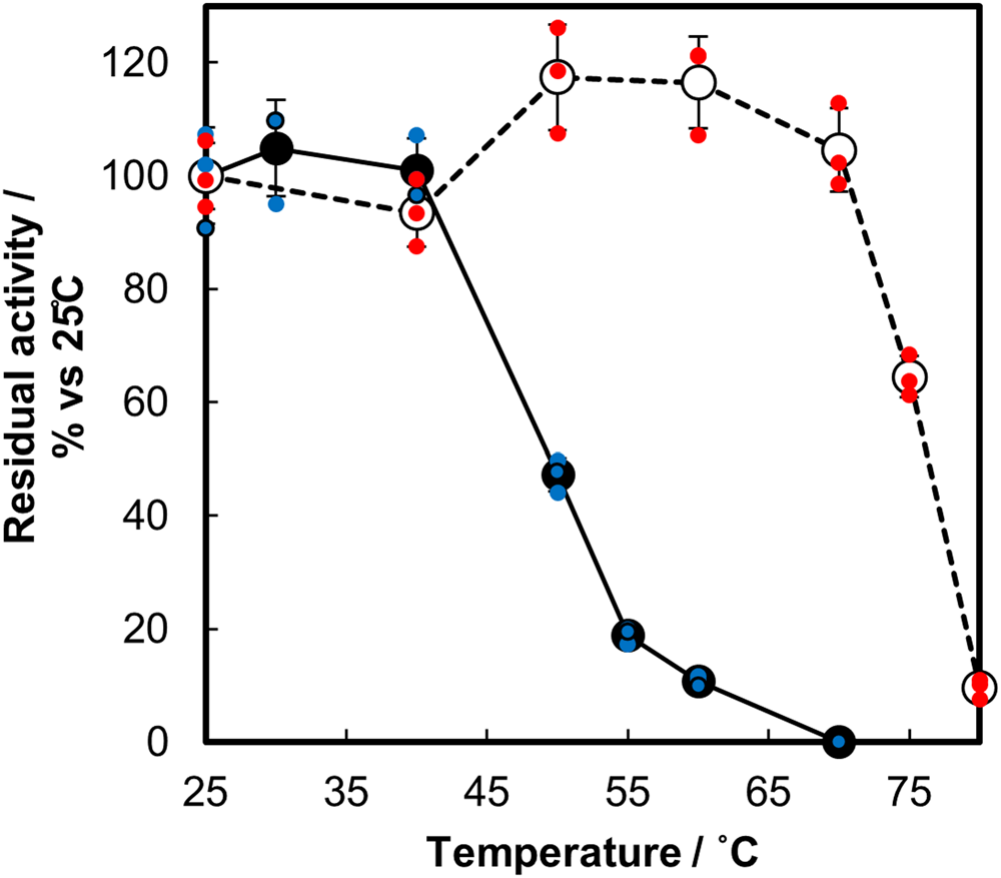
Thermal stability of the quaternary structure engineered BcGDH. Temperature dependence on residual enzymatic activity after 15 min. incubation is plotted. Mean values of residual activity are plotted as black closed circle and open circle for control (BcGDH without disulfide mutation) and αP205C/βD383C- γK155C/βY349C mutant BcGDHs, respectively. (*n* = 3, value ± SD) Data points for control enzyme or mutant enzyme are shown in blue or red small circles, respectively. Numerical Data for Fig. 3 can be found in Supplementary Data 1.

We then performed a detailed investigation of thermal stability for the quaternary structure-engineered BcGDH. Temperature-dependent inactivation experiments (Fig. 3; Numerical Data for Fig. 3 can be found in Supplementary Data 1) revealed that the multi-mutant enzyme was not inactivated at temperatures below 70 °C, and maintained activity even after incubation at 75 °C for 15 min; whereas, we observed partial inactivation of the BcGDH complex without additional disulfide bonds starting at 50 °C, and total inactivation within 3 min at 70 °C.

**Fig. 4:**
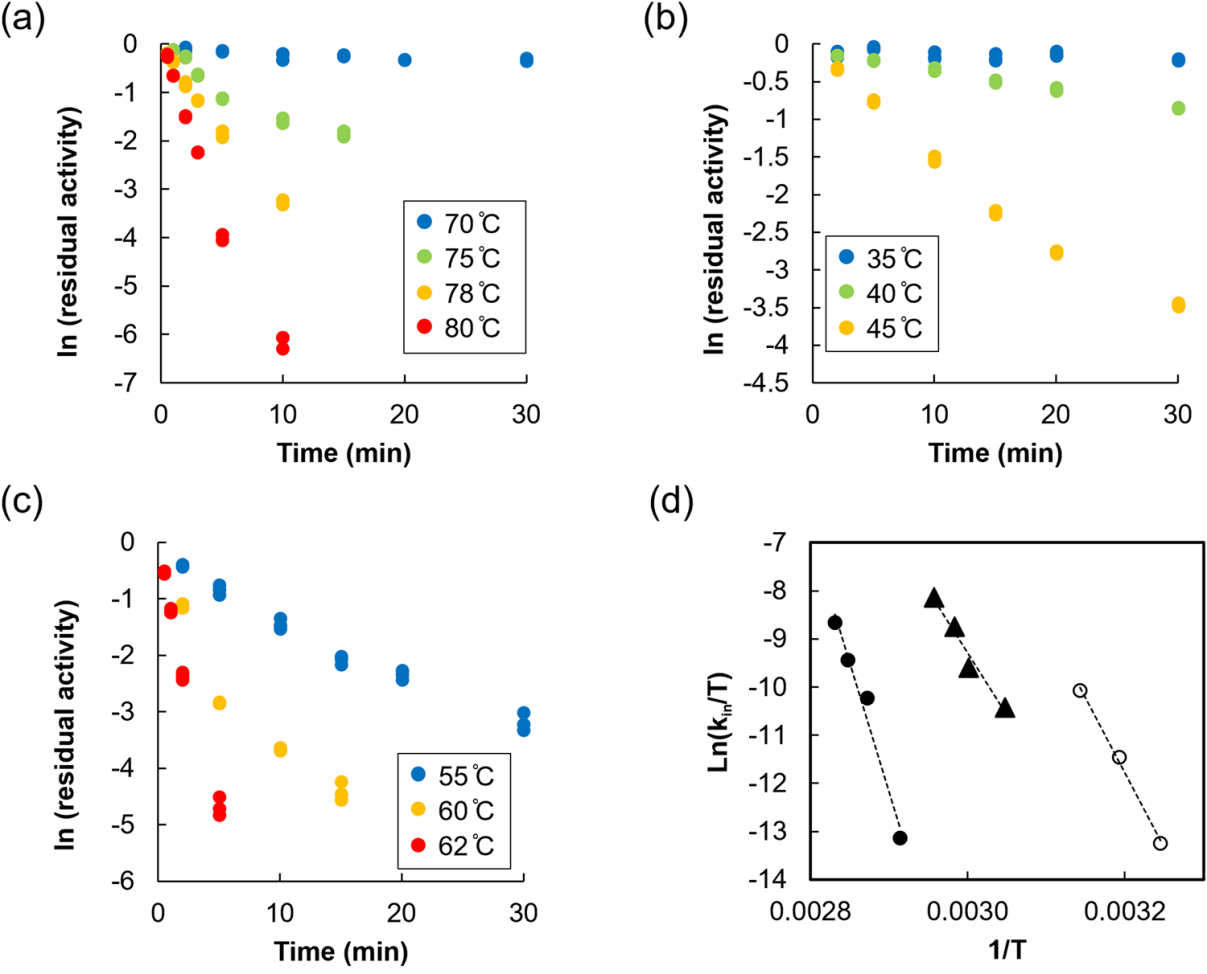
Thermal inactivation of the quaternary structure engineered BcGDH, BcGDH without additional disulfide bonds, and glucose oxidase derived from *Aspergillus niger* (AnGOx). **a**–**c** Time courses of thermal inactivation for **a** the quaternary structure engineered BcGDH, **b** BcGDH without additional disulfide bonds, and **c** glucose oxidase derived *from Aspergillus niger* (AnGOx) (*n* = 3). **d** Eyring plot analyses of thermal inactivation based on the first order rate constant of thermal inactivation (*k*_in_) obtained from (**a**–**c**). Numerical Data for Fig. 4a–d can be found in Supplementary Data 2.

We investigated thermal inactivation for the quaternary structure engineered BcGDH (Fig. 4a), BcGDH without additional disulfide bonds (Fig. 4b), and glucose oxidase derived from *Aspergillus niger* (AnGOx) (Fig. 4c) (Numerical Data for Fig. 4a–d can be found in Supplementary Data 2). The quaternary structure engineered BcGDH was incubated between 70 and 80 °C, whereas BcGDH without additional disulfide bonds between 35 and 45 °C, and GOx between 55 and 62 °C, considering the thermal stability of each enzyme. Through Eyring plot analyses (Fig. 4d, Table 3), ΔG^‡^ (45 °C) of the mutant was determined to be higher (129.6 kJ mol^−1^, ΔΔG^‡^ at 40.3 kJ mol^−1^) than the control BcGDH (89.3 kJ mol^−1^) and AnGOx (97.2 kJ mol^−1^). Similarly, ΔH^‡^ of the mutant enzyme (447.0 kJ mol^−1^, ΔΔH^‡^ at 187.9 kJ mol^−1^) was larger than the control BcGDH (259.1 kJ mol^−1^) and AnGOx (215.2 kJ mol^−1^). These results clearly indicate that introducing two inter-subunit disulfide bonds among catalytic/electron transfer and small/electron transfer subunits significantly improved enzyme stability. Furthermore, this enzyme proved to be more stable than GOx, and will not inactivate at physiologically relevant temperatures in solution.

**Table 3:** Calculated activated parameters of thermal inactivation from regression analysis of Eyring plot (45 ˚C).

	ΔH^‡^	ΔS^‡^	ΔG^‡^
	(kJ mol^−1^)	(kJ K^−1^ mol^−1^)	(kJ mol^−1^)
γK155C/βY349C- αP205C/βD383C	447.0	0.998	129.6
BcGDH control	259.1	0.533	89.3
AnGOx	215.2	0.371	97.2

### Electrochemical characterization

**Fig. 5:**
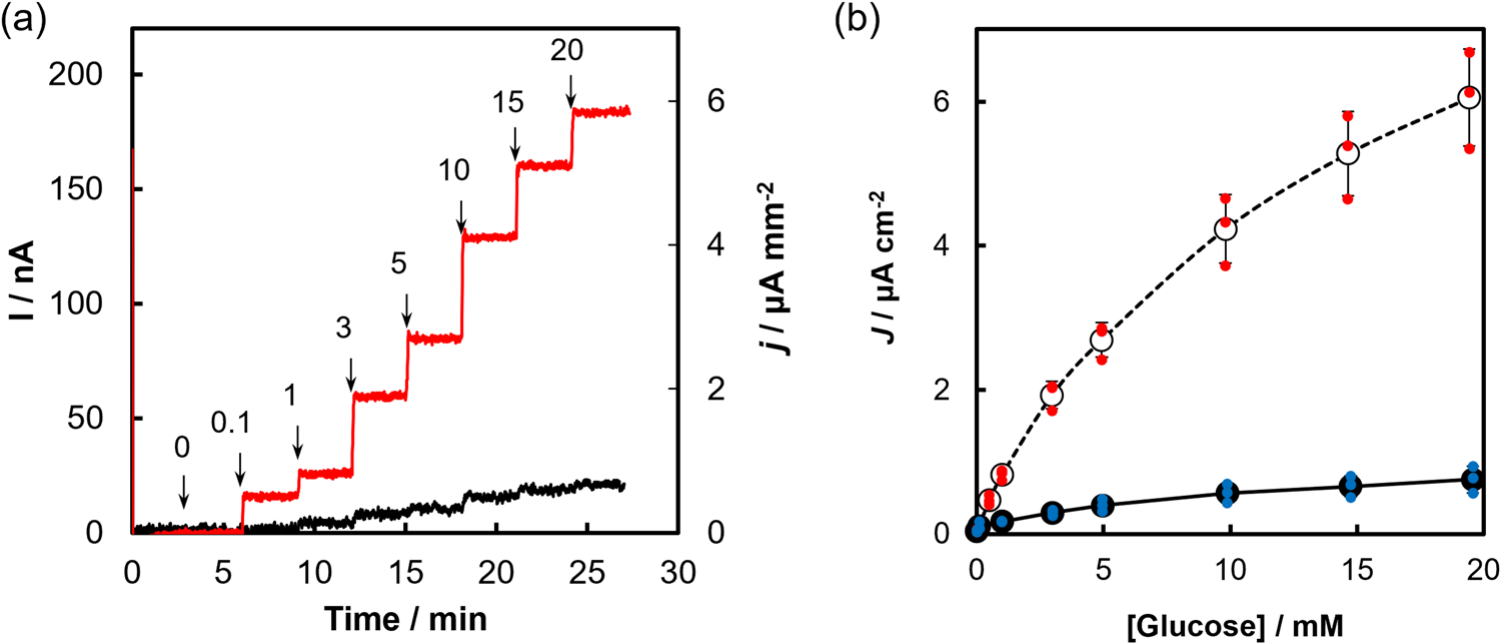
Amperometric evaluation of BcGDHs. BcGDH enzymes were modified on gold disk electrode (2 mmφ) and characterized as direct electron transfer type glucose sensors. Chronoamperometric evaluation of BcGDH immobilized enzyme electrode using DSH SAM at +200 mV (vs. Ag/AgCl) were carried out in 100 mM potassium phosphate buffer (pH 7.0). **a** Representative time course of amperometric measurements. Arrows and the numbers above indicate the addition point of glucose and its concentration (in mM). Time course of control and αP205C/βD383C- γK155C/ βY349C are shown as black and red lines, respectively. **b** Calibration curves of control and mutant BcGDH show as black closed circles and open circles, respectively. Evaluations were carried out in triplicate, and error bars indicate the respective standard deviation (*n* = 3, mean value ± SD). All data points for control or mutant enzyme are shown in blue or red small circles, respectively. Numerical Data for Fig. 5 can be found in Supplementary Data 3.

We characterized the DET ability of the quaternary structure engineered BcGDH immobilized by self-assembled monolayer (SAM) on gold rod electrodes in the absence of any additional electron mediators^11^. Representative time courses of amperometric measurements are shown in Fig. 5a (Numerical Data for Fig. 5a, b can be found in Supplementary Data 3). Following glucose addition, we immediately observed a current signal increase—approving DET and simultaneously indicating the introduction of two additional disulfide bonds did not impede nor impact the inherent DET ability of BcGDH. The current response increased in a glucose concentration-dependent manner (Fig. 5b), demonstrating that the constructed electrode with the quaternary structure engineered BcGDH can monitor glucose concentration within physiologically relevant hypo- (below 70 mg dL^−1^, or 3.9 mM), eu- (70–180 mg dL^−1^ or 3.9 mM–10 mM) and hyper- (higher than 180 mg dL^−1^ or 10 mM) -glycemic ranges. Furthermore, the current density derived from the constructed sensor was much higher than those fabricated with BcGDH without additional disulfide bonds, consistent with the improved catalytic efficiency of the engineered enzyme.

Overall, these results indicate that the quaternary structure engineered DET-type FADGDH, created through crystal structure information derived from space microgravity environments, is a promising candidate enzyme for future third-generation CGM glucose sensors.

## Discussion

In this study, we stabilized the DET-type FADGDH complex, BcGDH, through quaternary structure engineering by introducing disulfide bonds between the catalytic and electron transfer subunits, and between the small and electron transfer subunits. Disulfide bonds were designed based on the solved partial structure of this enzyme complex through preparing a protein crystal of BcGDH heterotrimer complex in space under JAXA PCG experiments, and by constructing a structural model showing the putative inter-subunit region. Our engineered BcGDH demonstrated improved thermal stability as well as increased catalytic and DET activity.

To elucidate the quaternary structure of BcGDH heterotrimer complex, we first investigated crystallization in laboratory conditions, but we could not obtain any crystals which yielded sufficient diffraction for structural analysis. Therefore, we joined the JAXA program to crystallize BcGDH. The effect of suppressing convection in liquid solution in microgravity crystallization has been reported by JAXA and others^19–24^. In microgravity environments, high-quality protein crystals without cracks caused by convection can be obtained which result in nice diffraction with low mosaicity for structural analysis. Many participants in JAXA’s program were able to obtain crystals with improved qualities and have reported structural analyses with higher resolution compared to their crystals grown on the ground.

Based on our structure model of the BcGDH complex obtained based on X-ray diffraction analyses, some salt bridges (βR315-γE134, βR315-αE50, and βR328-αE50) are likely formed in the N-terminal region of the β subunit (from βR315 to L331 colored with pink in Supplementary Fig. 4a). In addition, the N-terminal region of the β subunit likely forms hydrogen bonds with γ and α subunits between the carbonyl oxygen of βR315 and NH1 of γR78, the carbonyl oxygen of βV317 and ND2 of αN54, and the carbonyl oxygen of βL331 and NH1 of αR53. αP210 and αI224 of α subunit create a hydrophobic environment together with the hydrophobic residues of the β subunit (βP351, βL353, βV359, βL367, βV370, βI371, βV375, βP387, and βF389) and surround the third heme *c* (Supplementary Fig. 4b).

We previously reported a BcGDH mutagenesis study, which predicted the third heme *c* domain as the first electron acceptor in the electron transfer subunit from the catalytic domain via the iron-sulfur cluster as the electron donor^29^. This characteristic study also supports that the position of the third heme is structurally close to catalytic subunit. In addition to this, we characterized a BcGDH complex harboring a truncated electron transfer subunit, which only contains a third heme domain^30^. The truncated mutant retained full enzymatic and DET activity, suggesting the third heme is the first and only electron acceptor from the catalytic subunit. Taken together, these two biochemical observations indicate that the interaction between the third heme region in the electron transfer subunit and the other two subunits dominates the structural stability of an enzyme complex capable of DET. Accordingly, we reasoned that introducing disulfide bonds between the third heme region of the electron transfer subunit and the catalytic subunit and/or small subunit would enhance the quaternary structure necessary for DET.

We also predicted the structure of the BcGDH complex by AlphaFold-Multimer (Supplementary Fig. 5). The predicted structure revealed that only the third heme region in the electron transfer subunit interacts with the catalytic and small subunit.

Based on our biochemical data and the AlphaFold-Multimer structural prediction, we concluded our strategy to introduce disulfide bonds between the third heme region in the electron transfer subunit and the catalytic subunit and/or small subunit, was adequate, without considering additional interactions.

Our strategy to design and introduce disulfide bonds in the inter-subunit region was to avoid subunit dissociation while retaining its active quaternary structure demonstrating DET ability. The introduction of additional four cysteine residues in the selected mutant (αP205C/βD383C-γK155C/βY349C), did not negatively impact the catalytic activity (Table 2) nor the recombinant preparation of this enzyme compared to the control (Supplementary Table 3; mutant (αP205C/βD383C- γK155C/ βY349C); crude sample 3656 U/L culture, purified 282 U/L culture, control; crude sample 3092 U/L culture, purified 38 U/L culture). These results indicate that the additional disulfide bonds between subunits did not result in improper protein folding or an adverse structural change.

We believe our strategy is applicable for several enzyme complexes composed of multi-subunits, such as homologue enzyme complexes of BcGDH, fructose dehydrogenase (EC1.1.5.14), and 2-ketogluconate dehydrogenase (EC1.1.99.3). These enzymes form quaternary structures, and each is composed of a catalytic subunit harboring an FAD cofactor in its catalytic center with an iron sulfur cluster (Fe-S; 3Fe-4S) as the primary electron acceptor of FAD, a small subunit which is a hitch-hiker protein necessary for the bacterial TAT secretion system, and an electron transfer subunit harboring three heme *c* units.

The most stable BcGDH mutant, αP205C/βD383C-γK155C/βY349C, was not inactivated at temperatures below 70 °C and maintained activity even after incubation at 75 °C for 15 min. The mutant enzyme proved to have a higher thermal stability than the control BcGDH and the current commercially utilized enzyme, GOx. These results supported our hypothesis that the introduction of disulfide bonds within these three subunits enhances thermal stability. Our previous studies on introducing intra-molecular disulfide bonds in fungi-derived FADGDH resulted in values of 6.1 kJ mol^−1^ and 43 kJ mol^−1^ for ΔΔG^‡^ and ΔΔH^‡^, respectively^31^. Considering that introducing inter-subunit disulfide bonds resulted in a ΔΔG^‡^ value of 40.3 kJ mol^−1^ and a ΔΔH^‡^ value of 187.9 kJ mol^−1^, these results clearly indicate that the stabilization of quaternary structure through introducing two inter-subunit disulfide bonds, rather than intra-molecular disulfide bonds, significantly improved the enzyme stability.

The thermal stabilities and other enzymatic properties of reported thermally stable glucose dehydrogenases (GDHs) are summarized in Table 4. The most thermostable GDH was reported from a hyperthermophilic archaeon, *Thermoproteus* sp.^32^. In addition to this, several GDHs were reported from thermophilic bacteria and archeons^33–36^. However, these GDHs are NAD(P)-dependent enzymes which lack DET ability and are not suitable for the construction of continuous monitoring systems. Pyrroloquinoline quinone (PQQ)-bound GDHs were also reported from hypertheromophillic archeons^37^, however, these enzymes exhibit broad substrate specificity as well as show low catalytic efficiency towards glucose. We previously reported the quaternary structure engineering of PQQGDH derived from a mesophile (*Acinetobacter calcoaceticus*), which is a homo-dimeric enzyme. By introducing a disulfide bond in the inter-subunit region, hyper-thermal stabilization was achieved without loss of catalytic efficiency, which is comparable with this study. The improvement of substrate specificity^38^ and addition of DET ability of PQQGDH were also reported^39^, however, the combination of these engineering approaches is yet to be achieved to make PQQGDH suitable for CGM system. A limiting issue with PQQGDH is its redox cofactor. PQQ is not covalently bound to the enzyme and can form an apoenzyme during continuous operation. Fungi-derived thermostable enzymes have also been reported. Introducing intra-disulfide bonds to FADGDH enabled the construction of thermally stable enzyme. While several thermostable FADGDHs from fungi have been described, these FADGDHs do not have DET ability. Although a recent report outlined the construction of fungi-derived DET-FADGDH through the fusion of heme-*b* domains^40–42^, its DET abilities are inferior to the BcGDH. Therefore, among currently available glucose oxidoreductases, the quaternary structure engineered BcGDH is the most stable enzyme for use in CGM.

**Table 4:** The thermal stabilities and other enzymatic properties of reported thermally stable glucose dehydrogenases (GDHs).

EC	Organism	Km	*k*cat Vmax	melting temp (°C)	treatment temp (°C)	Halflife		Ref.
BcGDH wt								
1.1.5.9	*Burkholderia cepacia*				65	2.5 min	30 min, 10%	^5,18^
					65	72 min (crosslinked)		
*glucose 1-dehydrogenase [NAD(P)+]*								
1.1.1.47	*Thermoproteus tenax*		202 U mg^−1^ specific		103	10 min		^32^
	*Thermoplasma acidophilum*		320 U mg^−1^ specific		75	3 h	catalytic activity is retained for 9 h at 55 °C	^33^
	*Bacillus subtilis*	293 mM	720 s^−1^		70		30 min, 20%	^63^
	*Bacillus subtilis*	14 mM	395 s^−1^	50.2				^64^
	*Bacillus thuringiensis*	0.55 mM	11.4 U mg^−1^		65		30 min, > 85%	^65^
	*Bacillus thuringiensis*		93 U mg^−1^	72.2	65	8.0 min		^64^
	*Bacillus licheniformis*		0.05 U mg^−1^	70.3	65	8.7 min		^64^
	*Priestia megaterium*	11 / 63 mM (pH6 /8)	320 / 820 U mg^−1^		65		20 min, 20%	^66^
	*Priestia megaterium*	3.1 / 12 mM (pH6 /8)	230 / 810 U mg^−1^		65		40 min, 70%	^66^
	*Sulfurisphaera tokodaii*		32 U mg^−1^ specific		80		15 min, more than 90% remaining activity, pH 8.0	^34^
*glucose 1-dehydrogenase (NADP+)*								
1.1.1.119	*Haloferax mediterranei*	3.9 mM	550 U mg^−1^ specific		65	56.8 h (3 M NaCl)		^35^
*aldose 1-dehydrogenase (NAD(P)(+))*								
1.1.1.359	*Saccharolobus solfataricus*	8.0 mM(NAD) 0.44 mM (NADP)	437 U mg^−1^ specific		37	40 days		^36^
*glucose 1-dehydrogenase (PQQ, quinone)*								
1.1.5.2	*Pyrobaculum aerophilum*	680 mM	4.1 s^−1^		100		10 min, 100%	^37^
	*Sorangium cellulosum*	2.4 mM	1778 s^−1^		60		1 h, 27%	^67^
	*Arthrobacter globiformis*	0.21 mM	192 U mg^−1^		50		10 min, above 58%	^68^
	*Acinetobacter calcoaceticus*	24 mM	3300 U mg^−1^ 3436 s^−1^		55	9.5 min	wild-type isozyme PQQGDH-B: half-life 9.5 min	^69^
	*Acinetobacter calcoaceticus*	16 mM (S415C)	3461 s^−1^ (S415C)		70		10 min, 90% (mutant S415C)	^69^
	*Escherichia coli*	2.8 mM	116 U mg^−1^		51	10 min		^70^
*glucose 1-dehydrogenase (FAD, quinone)*								
1.1.5.9	*Thermoascus aurantiacus*	1.7 mM	~36.7 U mg^−1^		60		5 min, ~ 100%	^71^
	*Thermoascus crustaceus*	282 mM	~225 U mg^−1^	62.5	60		15 min, 75% (glycosylated)	^72^
	*Rasamsonia emersonii*	337 mM	~147 U mg^−1^	66.4	60		15 min, 89% (glycosylated)	^72^
	*Aspergillus terreus*	89.7 mM	541 U mg^−1^ specific		60	82 min		^73^

The catalytic current generated from the electrode with engineered BcGDH, αP205C/βD383C-γK155C/βY349C showed about 10 times higher catalytic current density than the control enzyme, which is much more than the ~2.5 times increase in the mutant’s catalytic activity (Table 2). An inherent issue of DET-type enzymes is the dissociation of the electron transfer subunit during the electrode immobilization process, which results in a loss of DET ability. Since the electron transfer subunit of control BcGDH may spontaneously dissociate during SAM based immobilization, the predicted amount of successfully immobilized BcGDH with an electron transfer subunit would be less than that of a BcGDH solution. The introduction of inter-subunit disulfide bonds in engineered BcGDH may prevent the dissociation of the electron transfer subunit during SAM formation and consequently improve the yield of active DET BcGDH on the surface of electrode. Therefore, we postulate the increase in catalytic current from the electrode functionalized with engineered BcGDH is due to the increased stability of the enzyme during immobilization.

Disulfide bond introduction resulted not only in increased thermal stability of BcGDH quaternary structure, but also increased catalytic activity. There are two possible explanations for this observation; the inter-subunit distance between the catalytic and electron transfer subunit is shortened, and inter-molecular electron transfer is facilitated. First, the introduction of disulfide bonds reduces the distance between the Fe-S cluster in the catalytic subunit and heme *c* in the electron transfer subunit. The crystal structure revealed the distance between the Fe-S cluster and heme *c* to be 7.9 Å (shortest distance: S_1_ in FeS to C_BC_ in heme). The criteria to select residues for cysteine substitution was based on the observation that the combination of residues which exist between catalytic/electron transfer subunits or small/electron transfer subunits is ~5 Å between β-carbons (C_β_s); 5.8 Å for αP205C/βD383C and 5.0 Å for γK155C/βY349C. However, these observed distances are the values before disulfide bond formation. Considering the average distances between β-carbons after disulfide bond formation are <5 Å^27,28^, formation of disulfide bonds between these residues might decrease the distance and fluctuation in distance between catalytic/electron transfer subunits and/or small/electron transfer subunits, bringing the Fe-S cluster in the catalytic subunit and heme *c* in the electron transfer subunit in closer proximity, consequently improving electron transfer efficiency and catalytic activity. Marcus theory^43^ states the inverse of distance contributes to the reorientation energy, and the reorientation energy exponentially affects the electron transfer rate, suggesting that the effect of shortening the distance between redox centers on electron transfer is high.

The second possibility is the role of these newly formed disulfide bonds; they may serve as electron acceptors of the Fe-S cluster. It has been reported that the disulfide ring formed by adjacent cysteine residues in quinoprotein ethanol dehydrogenase (QEDH) serves an essential role for electron transfer from its cofactor pyrroloquinoline quinone (PQQ) to cytochrome *c*_550_, the natural electron acceptor of this enzyme^44–46^. PQQ complexed with a Ca^2+^ ion is located in the superbarrel center and is embedded between a tryptophan residue and a ring structure formed from a disulfide bridge between adjacent cysteines Cys105 and Cys106^47^. This disulfide ring near PQQ is found in all quinoprotein and quinohemoprotein alcohol dehydrogenases and is essential for enzyme activity. Recently, redox activity of the disulfide ring in quinoprotein methanol dehydrogenase was confirmed, more specifically through its participation with the PQQ prosthetic group in electron transfer within the protein^48^. Considering the observations that disulfide bonds existing close to redox cofactors can serve as electron acceptors or electron donors, the newly formed disulfide bond in BcGDH, most probably in αP205C/βD383C, might serve as an additional redox-active molecule which facilitates electron transfer from the Fe-S cluster in the catalytic subunit and heme c in the electron transfer subunit and increases catalytic efficiency.

To consider BcGDH as a sensor constituent in CGM systems, the enzyme should maintain activity and specificity in complex biological samples. We have previously reported that enzyme electrodes employing BcGDH worked well not only in buffer solutions, but also in biological matrices utilized in continuous monitoring, such as artificial interstitial fluid (ISF)^49^ and artificial sweat^49,50^. It is also important to note that we utilized an engineered BcGDH with improved substrate specificity in this study, and the introduction of disulfide bonds did not negatively impact specificity (Supplementary Table 4). In addition, the mutations introduced in this study were not deleterious to the enzyme’s catalytic properties. Taken together, these observations suggest a similar electrochemical performance of mutant BcGDH in pseudo-biological samples as those reported in buffer.

Introducing inter-subunit disulfide bonds within BcGDH stabilized its quaternary structure and resulted in an enzyme with high thermal stability. Furthermore, mutants constructed with two additional disulfide bonds demonstrated superior catalytic activity and catalytic current with electrochemical measurement. BcGDH is a highly remarkable enzyme in the biosensing field due to its DET ability. This mutant enzyme which exhibits high stability and high DET ability shows decided promise for application in future glucose sensing devices. Furthermore, this strategy for enzyme stabilization can be applied to other DET type enzymes.

## Methods

### Preparation of enzyme: recombinant expression and purification

Recombinant BcGDHs were prepared using expression vector pTrc99A containing the structural gene for BcGDH and pBBJMccm for heme *c* maturation^7,29^. The sequences of enzymes are shown in Supplementary Data. These vectors were co-transformed into *Escherichia coli* strain BL21(DE3) and cultivated according to our previous reports^16,29^. Co-transformed *E. coli* were cultured in 500 mL conical flasks containing 100 mL of ZYP-5052 medium^51^ in a rotary shaker at 30 °C for 30 h. Cells were harvested by centrifugation.

Harvested wet cells were disrupted by sonication in 20 mM Tris-HCl buffer pH 7.8 containing 0.2% Triton X-100. The lysate was centrifuged at 16,000 x*g* for 20 min and the supernatant was then dialyzed overnight against 20 mM Tris-HCl buffer pH 7.8 containing 0.1% Triton X-100. The dialyzed sample was applied to HiTrap Q HP column (Cytiva) equilibrated with 20 mM Tris-HCl, pH 7.8 containing 0.1% Triton X-100. Protein was eluted with a linear NaCl gradient in same buffer. The active fraction was pooled and dialyzed overnight at 4 °C against PBS containing 0.1% Triton X-100 and applied to Superdex-200 increase 10/300GL column (Cytiva) equilibrated with PBS containing 0.1% Triton X-100. Pooled fractions were then dialyzed at 4 °C against 10 mM potassium phosphate buffer containing 0.1% Triton X-100, pH 7.0. Purity of purified protein was confirmed by SDS-PAGE (Supplementary Fig. 2a; uncropped and unedited gel images are shown in Supplementary Fig. 2b).

For crystallization, recombinant expression of BcGDH was done following the previous method except we utilized a BcGDH expression plasmid which contained a His-tag at the C-terminal of the electron transfer subunit. Harvested cells were disrupted using a French pressure. The membrane fraction was prepared by centrifugation of cell lysates, followed by ultracentrifugation. The resulting membrane fraction was solubilized in a 10 mM potassium phosphate buffer (PPB), pH 7.0 containing 1.0% (w/v) n-dodecyl-β-D-maltoside (DDM) and crude enzyme was prepared from supernatant after ultracentrifugation. Crude enzyme sample was applied to HisTrap HP column (Cytiva) equilibrated with 10 mM PPB pH 7.0 containing 20 mM Imidazole, 0.5 M NaCl, 0.03% DDM. Protein was eluted with a linear imidazole gradient in same buffer. The active fraction was pooled and dialyzed overnight at 4 °C against 10 mM P.P.B. containing 0.03% DDM and applied to HiTrap Q HP column (Cytiva) equilibrated with 10 mM PPB containing 0.03% DDM. Protein was eluted with a linear NaCl gradient in same buffer. Pooled fractions were then dialyzed at 4 °C against 10 mM PPB pH 7.0.

### Crystallization

Crystals of BcGDH were grown by utilizing the crystallization facilities of the JAPAN Aerospace Exploration in the Japanese Experiment Module “Kibo” at the International Space Station (ISS) under a high-quality protein crystal growth project (JAXA-PCG). In a microgravity environment at the ISS, the crystals were grown by counter-diffusion with the protein solution (5 mg/ml in 10 mM potassium phosphate, pH 7.0, 0.03% (w/v) DDM) in a reservoir containing 2% (v/v) tacsimate, 16% (w/v) PEG 3350, 0.03% (w/v) DDM and 100 mM sodium citrate, pH 5.6 using the JCB-SGT device^52,53^. The obtained crystals were flash-frozen and stored in liquid nitrogen.

### X-ray crystallography

X-ray diffraction data of a crystal of BcGDH were collected at 100 K using a PILATUS3 6 M detector at a wavelength of 1.0 Å on the BL41XU beam line in the SPring-8 (Hyogo, Japan). Diffraction data were processed using XDS^54^ and CCP4 program suite^55^. The initial phases of BcGDH were obtained by molecular replacement using the program MOLREP^56,57^ with the structure of BcGDH small/catalytic subunits complex (PDB code: 6a2u). Although the electron density maps of a complex of BcGDH small/catalytic subunits and a part of the electron transfer subunit that attaches to BcGDH small/catalytic subunits complex are visible, the entire electron transfer subunit was not visible due to the weak and ambiguous electron density.

In the analysis of sequence similarity of electron transfer subunit of BcGDH by blast search, C-terminal region (Leu359-Arg456) of heme-Cu nitrite reductase from *Ralstonia pickettie* was raised up showing the similarity to the C-terminal 3rd heme *c* region (Leu329 to Arg425) of electron transfer subunit of BcGDH with 31% identity. We used the structure information of the C-terminal region of heme-Cu nitrate reductase (PDB ID: 4AX3) and also used the predicted model by Phyre2^58^ (which was also predicted by using 4AX3) for the second molecular replacement as search model for a partial electron transfer subunit after initial phase determination.

A partial model building of the electron transfer subunit was then performed in the program Coot^59^, and the structure was refined using the program Refmac5^60,61^. We used this partial constructed model of the electron transfer subunit for further investigation of engineering BcGDH. The finally refined structure was deposited in the Protein Data Bank (PDB) as 8HDD. In Ramachandran plots, 80.7% and 10.9% of all residues in BcGDH were shown to be preferred regions and allowed regions, respectively. The rest 8.4% was outliers. Data collection and refinement statistics are listed in Table 5.

**Table 5:** Data collection and refinement statistics (molecular replacement).

	BcGDH
Data collection	
Space group	*P*2_1_2_1_2
Cell dimensions	
*a*, *b*, *c* (Å)	204.01, 71.80, 114.22
Resolution (Å)	50.00–3.0 (3.08–3.0)^a^
*R*_merge_^b^	0.165 (0.732)
*I* / σ*I*	10.3 (3.0)
Completeness (%)	99.8 (96.1 l)
Redundancy	6.7 (7.0)
Refinement	
Resolution (Å)	49.88–3.0
No. reflections	32619
*R*_work_ / *R*_free_	0.278 / 0.323
No. atoms	
Protein	5935
Ligand/ion	103
* B*-factors	
Protein	85.9
Ligand/ion	76.1
R.m.s. deviations	
Bond lengths (Å)	0.002
Bond angles (°)	1.135

^a^Values in parentheses are for highest-resolution shell.

^b^*R*_merge_ = Σ_*hkl*_ Σ_*i*_ [|*I*_*i*_(*hkl*) - <*I*(*hkl*)>|/ Σ_*hkl*_ Σ_*i*_ *I*_*i*_(*hkl*)], where *I*_*i*_(*hkl*) is the intensity value of the *i*th measurement of reflection *hkl* and <*I*(*hkl*)> is the mean value of *I*_*i*_(*hkl*) for all *i* measurements.

### Enzymatic assay

The activities of crude extracts and the purified recombinant BcGDH were determined using methods described in a previous study^6,62^ with slight modifications based on either the PMS/DCIP or Ru/MTT system. For the PMS/DCIP method, the enzyme sample was incubated in 20 mM potassium phosphate buffer (pH 7.0) containing 0.6 mM 5-methylphenazinium methylsulfate (PMS), 0.06 mM 2,6-dichlorophenolindophenol (DCIP), and glucose. The activity was determined by monitoring the decrease in absorbance from DCIP at 600 nm and using the molar absorption coefficient of DCIP (16.3 mM cm^−1^ at pH 7.0) to calculate enzyme activity. For the Ru/MTT method, the enzyme sample was incubated in 20 mM potassium phosphate buffer (pH 7.0) containing 77 mM hexaammineruthenium (III) chloride, 1 mM 3-(4,5-di-methylthiazol-2-yl)-2,5-diphenyltetrazolium bromide and 100 mM glucose. The activity was determined by monitoring the increase in absorbance from formazan at 565 nm and using the molar absorption coefficient of formazan (20 mM cm^−1^ at pH 7.0). In each measurement method, one unit of enzyme activity is defined as the amount of enzyme that oxidizes 1 μmol of glucose per min.

The thermal stability of each enzyme was determined by incubating the enzymes (0.45 mg mL^−1^ for crude enzyme, 0.1 or 0.4 mg protein mL^−1^ for purified enzyme) in 20 mM potassium phosphate buffer (pH 7.0) at various temperatures (25–80 °C). After heat treatment, samples were placed on ice for at least two minutes and residual activity measurements were done as described above.

Inactivation parameters were obtained based on transition state theory according to a previous report^31^. First order inactivation rate constants *k*_in_ at temperature *T* were calculated from the time-dependence of inactivation at each temperature. Then, Eyring plot analysis ln(*k*_in_/*T*) vs. 1/*T*, was carried out. Activation free energy Δ*G*^‡^ and activating parameters Δ*H*^‡^ and Δ*S*^‡^ were calculated using following Eqs. 1 and 2:

1 $${{{{{\rm{ln}}}}}}\left(\frac{{k}_{{{{{{\rm{in}}}}}}}}{T}\right)={{{{{\rm{ln}}}}}}\left(\frac{{k}_{{{{{{\rm{B}}}}}}}}{h}\right)+\frac{\triangle {S}^{{{\ddagger}} }}{R}-\frac{\triangle {H}^{{{\ddagger}} }}{{RT}}$$

2 $$\triangle {G}^{{{\ddagger}} }=-{RT}{{{{{\rm{ln}}}}}}\left(\frac{{k}_{{{{{{\rm{in}}}}}}}h}{{k}_{{{{{{\rm{B}}}}}}}T}\right)$$

Where *T* is the absolute temperature in K; *k*_B_ (J K^−1^) and *h* (J s) are the Bolzmann’s and Planck’s constant; R (J K^−1^ mol^−1^) is the universal gas constant.

### Electrochemical measurement

2 mm diameter gold disk electrodes (GDEs, CH instruments) were utilized to construct the enzyme modified electrode. GDE surfaces were polished and cleaned with piranha solution (H_2_SO_4_:30% H_2_O_2_ = 3:1) for 15 min. To form the self-assembled monolayer (SAM) on the electrode surface, electrodes were soaked in 100 μM dithiobis succinimmidyl hexanoate (DSH) solution in acetone for 24 h following cleaning^11^. The DSH-SAM modified GDEs were rinsed with acetone and incubated in 0.05 mg/mL enzyme solution in 20 mM P.P.B. (pH 7.0) at 4 °C. The enzyme-functionalized electrodes were stored in 100 mM P.P.B. (pH 7.0) until use.

Amperometric measurements were conducted with the enzyme modified working electrodes, and an Ag/AgCl (CH111, CH instruments) and Pt wire (Tanaka precious metals) reference and counter electrode, respectively. A bias potential of +200 mV (vs. Ag/AgCl) was applied, and the generated current was recorded using an electrochemical analyzer (VSP-3, BioLogic). Chronoamperometry was carried out in 100 mM potassium phosphate buffer (pH 7.0) at room temperature.

### Statistics and reproducibility

Enzymatic assay and electrochemical measurements were conducted in triplicate. Acquired data are presented as the mean values ± standard deviation (SD).

### Reporting summary

Further information on research design is available in the Nature Portfolio Reporting Summary linked to this article.

## Supplementary information

Supplementary Materials

Description of Additional Supplementary Files

Supplementary Data 1-5

Reporting Summary

## Data Availability

The coordinate of the refined structure was deposited in Protein Data Bank as PDB ID (8HDD). All data needed to evaluate the conclusions in the paper are present in the paper and/or the Supplementary Materials and Supplementary Data. Source data for figures can be found in Supplementary Data 1–3.
