# Supplementary material for: Microgravity environment grown crystal structure information based engineering of direct electron transfer type glucose dehydrogenase
Source: Commun Biol. 2022 Dec 6;5:1334. doi: 10.1038/s42003-022-04286-9 (PMC9727119; doi:10.1038/s42003-022-04286-9)
Supplement: Supplementary file 1 — Supplementary Materials [file 42003_2022_4286_MOESM1_ESM.pdf]

Supplementary Materials for

Microgravity environment grown crystal structure information based engineering of direct electron transfer type glucose dehydrogenase

Junko Okuda-Shimazaki[1†], Hiromi Yoshida[2†], Inyoung Lee[1], Katsuhiro Kojima[3], Nanoha Suzuki[3], Wakako Tsugawa[3], Mitsugu Yamada[4], Koji Inaka[5], Hiroaki Tanaka[6], Koji Sode[1]*

*Corresponding author. Email: ksode@email.unc.edu

† These authors contributed equally to this work

This PDF file includes:

Supplementary Tables 1 to 4
Supplementary Figures 1 to 5
Supplementary References 1-6

Supplementary Table 1. Estimated distances between amino acid pairs for disulfide bond formation

	C_β-C_β Å	C_α-C_α Å
αW46-βL329	6.4	7.0
αW46-βN333	3.6	4.5
αP205-βD383	5.8	7.2
αG208-βG385	-	9.3
αP210-βP387	4.8	5.9
αN215-βT336	5.8	7.4
αP223-βT336	4.2	6.9
αI224-βT336	6.4	7.8
αE235-βY391	5.8	8.3
γT145-βP346	5.5	6.7
γY151-βT336	5.2	4.1
γY151-βQ339	5.4	6.3
γN154-βT345	5.0	5.1
γK155-βY349	5.0	6.5

Supplementary Table 2. Relative expression level of catalytic subunits in crude enzyme sample

Crude sample	Relative expression vs. control
1. Control	100
2. αP205C/βD383C-γK155C/βY349C	44
3. αP205C/βD383C	57
4. γK155Cβ/Y349C	65
5. αE235C/βY391C	48
6. γT145C/βP346C	75
7. αW46C/βN333C	30
8. αW46C/βL329C	77
9. αG208C/βG385C	32
10. αP210C/βP387C	32
11. αN215C/βT336C	78
12. αP223C/βT336C	21
13. αI224C/βT336C	37
14. γY151C/βT336C	50
15. γY151C/βQ339C	47
16. γN154C/βT345C.	45

Catalytic subunit expression levels in crude enzyme samples were analyzed by scanning the SDS-PAGE gel (Figure S1) in ImageJ (*1-3*). Band intensities corresponding to the catalytic subunit for each enzyme sample was normalized by internal control. The relative intensity for each mutant sample was compared with the control enzyme sample being 100%.

Supplementary Table 3. Production yield throughout Purification

	Fraction	U/L culture	Yield (%)	U/mg	Purification fold
**Control**	After sonication	3092	100	**2.38**	**1**
	Soluble fraction	1927	62.3	**1.75**	**0.74**
	Anion exchange	125	4.04	**24.20**	**10.18**
	Purified	38	1.23	**126.56**	**53.21**
**αP205C/βD383C-γK155C/ βY349C**	After sonication	3656	100	**4.68**	**1**
	Soluble fraction	3070	84.0	**4.37**	**0.93**
	Anion exchange	985	27.0	**60.2**	**12.85**
	Purified	282	7.73	**281**	**59.94**

Supplementary Table 4. Substrate specificity

Relative specific activity (%) vs specific activity with glucose as 100%

**Control**		Gal	Xyl	Mal
	50 mM	105	1.31	1.31
	5 mM	98.5	0.26	0.34
**αP205C/βD383C-γK155C/ βY349C**		Gal	Xyl	Mal
	50 mM	106	3.78	1.13
	5 mM	92.0	0.08	0.15

Substrate specificity of each enzyme was compared by relative specific activity. Activity measurements were carried out with Ru/MTT system using 50 mM or 5 mM of each substrate (galactose, xylose or maltose). Disulfide bond introduction did not affect substrate specificity of this enzyme.

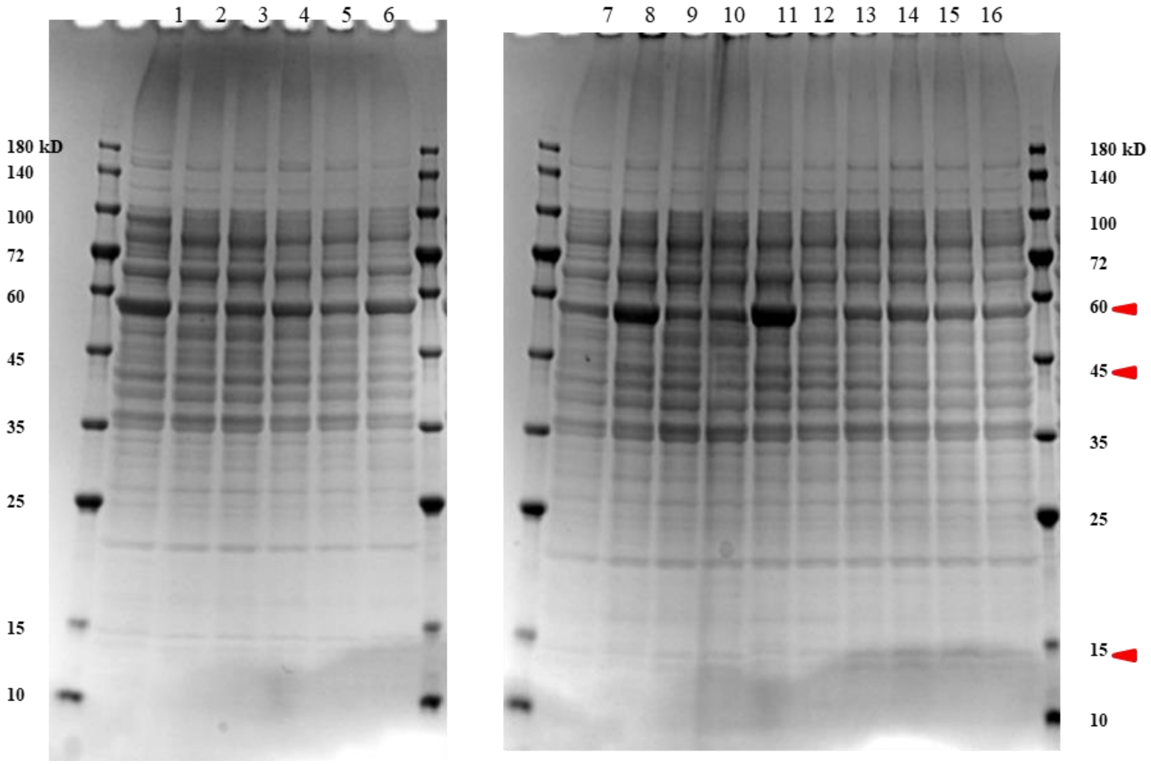

Supplementary Figure 1a. SDS-PAGE analysis of the crude extracts of recombinantly prepared BcGDH inter-subunit disulfide bond-introduced mutants.

Major bands (red arrows) corresponding to the size of catalytic (60 kDa), electron transfer (43 kDa) and small subunit (13 kDa) domains were observed. Lane labels correspond to: 1. Control, 2. αP205C/βD383C-γK155C/βY349C, 3. αP205C/βD383C, 4. γK155Cβ/Y349C, 5. αE235C/βY391C, 6. γT145C/βP346C, 7. αW46C/βN333C, 8. αW46C/βL329C, 9. αG208C/βG385C, 10. αP210C/βP387C, 11. αN215C/βT336C, 12. αP223C/βT336C, 13. αI224C/βT336C, 14. γY151C/βT336C, 15. γY151C/βQ339C, 16. γN154C/βT345C.

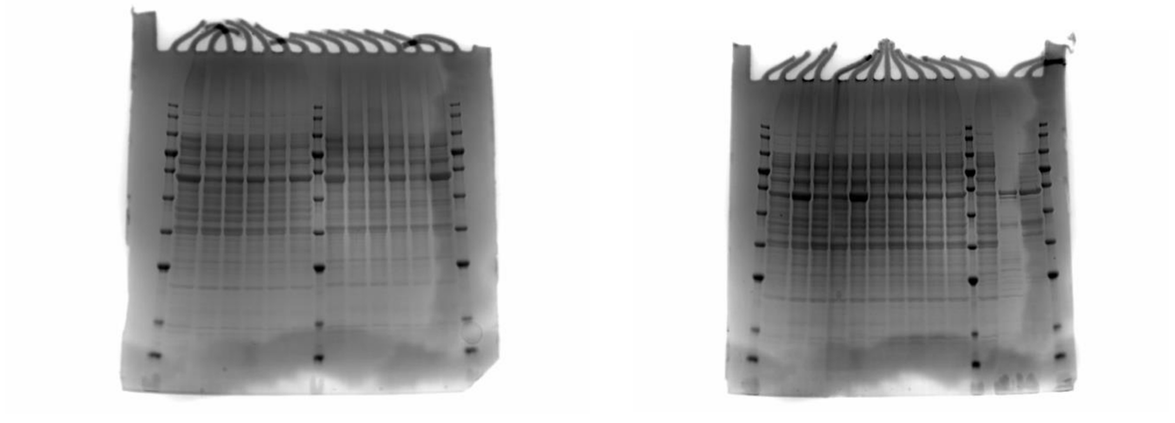

Supplementary Figure 1b. Uncropped/unedited SDS-PAGE gel images of Supplemented Figure 1a

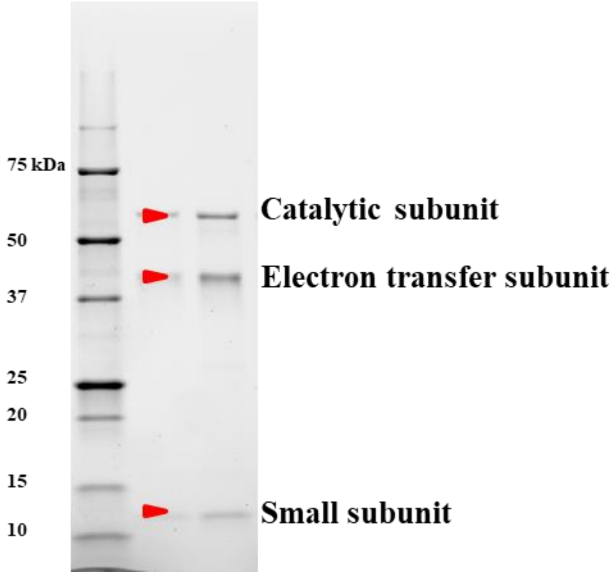

Supplementary Figure 2a. SDS-PAGE of purified BcGDH αP205C/βD383C- γK155C/ βY349C enzyme

Three major bands corresponding to catalytic, electron transfer, and small subunit were observed in the purified enzyme after size exclusion chromatography. The calculated size of each subunit is as follows; the catalytic subunit is 60 kDa from 540 a.a., the electron transfer subunit is 43 kDa from 401 a.a, and the small subunit is 13 kDa from 121 a.a.

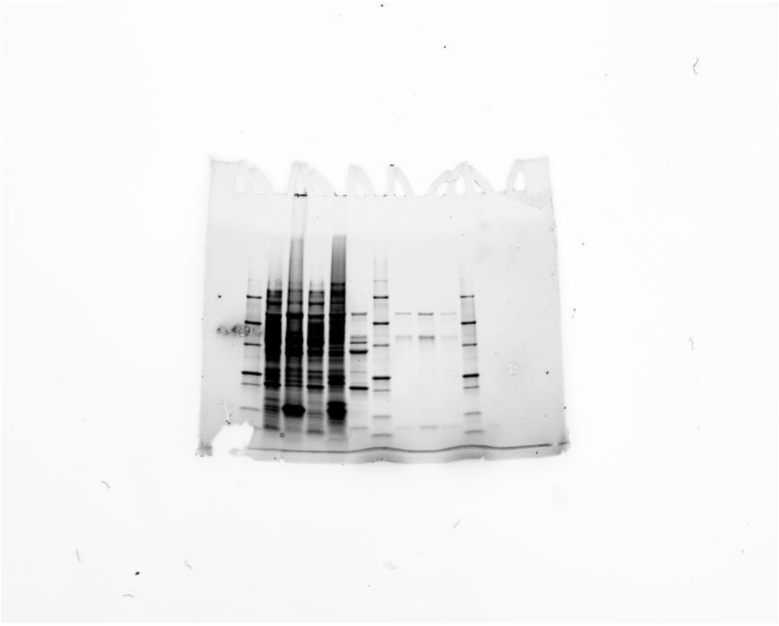

Supplementary Figure 2b. Uncropped/unedited SDS-PAGE gel images of Supplemented Figure 2a

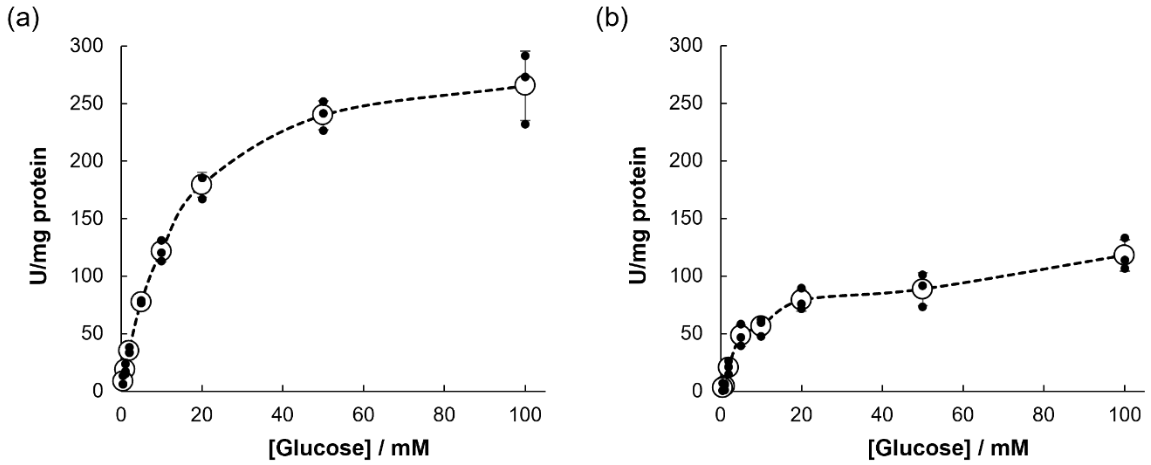

Supplementary Figure 3. Glucose-dependent enzyme activity of purified enzyme samples.
Glucose concentration dependence of specific activities of (a) BcGDH inter-subunit disulfide bond-introduced mutant (αP205C/βD383C-γK155C/βY349C) and (b) control. The specific activity of purified enzyme was plotted depending on the glucose concentration. Specific activities were measured by Ru/MTT system. Each data point is depicted by closed circle and mean value is shown in open circle. (n=3, ±SD). Numerical Data for Supplementary Figure 3 can be found in Supplementary Data 4.

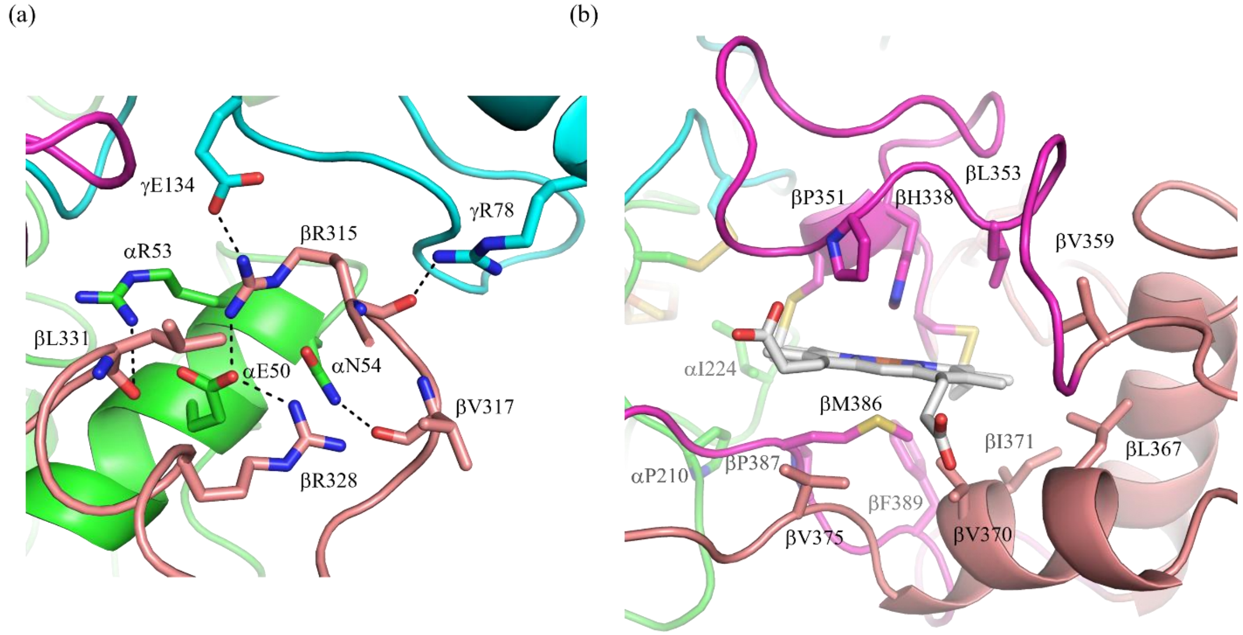

Supplementary Figure 4. The inferred interactions at the subunit interface.

(a) The salt bridge and hydrogen bonds at the subunit interface. (b) The hydrophobic environment around third heme *c*. Not only the hydrophobic residues of the β-subunit, but also P210 and I224 of the α-subunit create the hydrophobic environment. These interactions were inferred from the solved structure (PDB ID 8HDD). Dotted lines show the predicted salt bridge and hydrogen bonds in the model. α denotes the catalytic subunit, γ denotes the small subunit, and β denotes the electron transfer subunit, colored with green, cyan, pink respectively. The third heme *c* is shown in a white stick model.

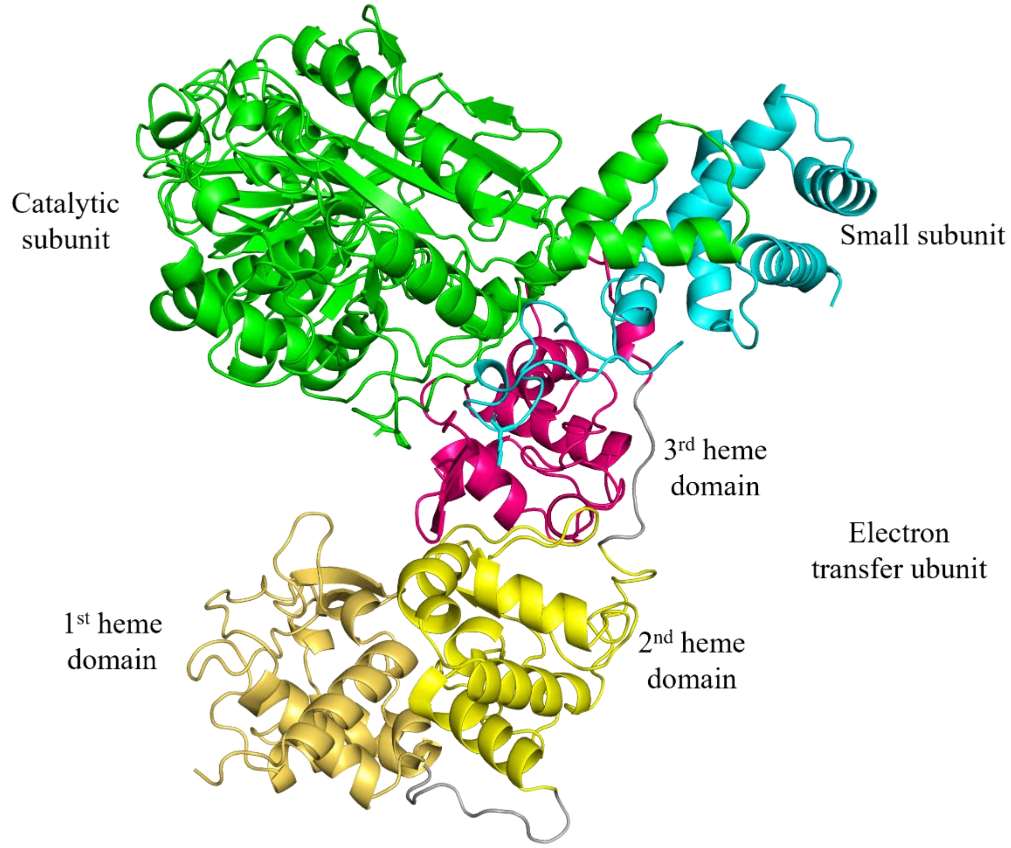

Supplementary Figure 5. Predicted structure of a BcGDH heterotrimer complex by AlphaFold-Multimer

Structure model of the heterotrimer complex of BcGDH was constructed by AlphaFold multimer (4-6). The model structure is well-fitted to the crystal structure acquired in this study (RMSD:0.520).
Each subunit is colored as follows: catalytic subunit (green), small subunit (cyan), first heme domain of the electron transfer subunit (orange), second heme domain (yellow), third heme domain (magenta).

Supplementary References

1. W.S. Rasband, ImageJ, U. S. National Institutes of Health, Bethesda, Maryland, USA, http://rsb.info.nih.gov/ij/, 1997-2012.
2. C.A. Schneider, W.S. Rasband, K.W. Eliceiri, NIH Image to ImageJ: 25 years of image analysis. *Nature Methods* 9, 671-675, (2012)
3. M.D. Abramoff, P.J. Magelhaes, S.J.Ram, Image Processing with ImageJ. *Biophotonics International*, **11**, 36-42, (2004)
4. J. Jumper et al. Highly accurate protein structure prediction with AlphaFold. Nature, 596(7873):583–589, 2021. DOI: 10.1038/s41586-021-03819-2
5. Usman Ghani et al., Improved docking of protein models by a combination of AlphaFold2 and ClusPro. bioRxiv, 2021. doi: https://doi.org/10.1101/2021.09.07.459290
6. Richard Evans et al., Protein complex prediction with AlphaFold-Multimer, ClusPro. bioRxiv, doi: https://doi.org/10.1101/2021.10.04.463034
