# Supplementary material for: Microgravity environment grown crystal structure information based engineering of direct electron transfer type glucose dehydrogenase
Source: Commun Biol. 2022 Dec 6;5:1334. doi: 10.1038/s42003-022-04286-9 (PMC9727119; doi:10.1038/s42003-022-04286-9)
Supplement: Supplementary file 2 — Description of Additional Supplementary Files [file 42003_2022_4286_MOESM2_ESM.docx]

**Description of Additional Supplementary Files**

**File name:** Supplementary Data 1-5
**Description:**

Supplementary Data 1: Numerical Data for Figure 3 (Residual activity vs. 25 ̊C, n=3).

Supplementary Data 2: Numerical Data for Figure 4a-d (Ln (residual activity), n=3).

Supplementary Data 3: Numerical Data for Fig. 5.

Supplementary Data 4: Numerical Data for Supplementary Figure 3.

Supplementary Data 5:
