# Supplementary material for: Microgravity environment grown crystal structure information based engineering of direct electron transfer type glucose dehydrogenase
Source: Commun Biol. 2022 Dec 6;5:1334. doi: 10.1038/s42003-022-04286-9 (PMC9727119; doi:10.1038/s42003-022-04286-9)
Supplement: Supplementary file 3 — Supplementary Data 1-5 [file 42003_2022_4286_MOESM3_ESM.pdf]

Supplementary Data for

Microgravity environment grown crystal structure information based engineering of direct electron transfer type glucose dehydrogenase

Junko Okuda-Shimazaki[1†], Hiromi Yoshida[2†], Inyoung Lee[1], Katsuhiro Kojima[3], Nanoha Suzuki[3], Wakako Tsugawa[3], Mitsugu Yamada[4], Koji Inaka[5], Hiroaki Tanaka[6], Koji Sode[1]*

*Corresponding author. Email: ksode@email.unc.edu

† These authors contributed equally to this work

Supplementary Data 1-4

Sequences of enzymes

Supplementary Data 1

Numerical Data for Figure 3 (Residual activity vs. 25℃, n=3)

	Temp	#1	#2	#3	Mean
control	25	90.63	107.37	102.00	100.00
	30	109.66	109.85	95.07	104.86
	40	96.45	99.29	107.16	100.97
	50	47.75	49.79	44.10	47.21
	55	19.40	19.87	17.23	18.83
	60	9.87	11.57	10.75	10.73
	70	0.07	0.05	0.00	0.04
mutant	25	99.29	94.52	106.18	100.00
	40	99.31	93.36	87.46	93.38
	50	107.56	126.13	118.51	117.40
	60	121.22	107.15	121.07	116.48
	70	102.23	98.54	112.88	104.55
	75	61.24	63.60	68.43	64.43
	80	10.91	10.16	7.49	9.52

Supplementary Data 2

Numerical Data for Figure 4a-d (Ln (residual activity), n=3)

Mutant (Fig.4a)						Control (Fig.4b)					
		Ln(residual activity)						Ln(residual activity)			
temp	time (min)	#1	#2	#3	Mean	temp	time (min)	#1	#2	#3	Mean
70	2	-0.066	-0.130	-0.092	-0.096	35	2	-0.127	-0.172	-0.097	-0.132
	5	-0.134	-0.162	-0.139	-0.145		5	-0.075	-0.050	-0.033	-0.052
	10	-0.200	-0.210	-0.326	-0.244		10	-0.171	-0.103	-0.196	-0.156
	15	-0.243	-0.258	-0.219	-0.240		15	-0.195	-0.218	-0.127	-0.179
	20	-0.325	-0.323	-0.324	-0.324		20	-0.095	-0.143	-0.155	-0.131
	30	-0.353	-0.337	-0.289	-0.326		30	-0.199	-0.222	-0.218	-0.213
75	1	-0.111	-0.126	-0.142	-0.126	40	2	-0.151	-0.147	-0.153	-0.150
	2	-0.277	-0.261	-0.239	-0.259		5	-0.223	-0.227	-0.208	-0.219
	3	-0.619	-0.645	-0.662	-0.642		10	-0.314	-0.355	-0.362	-0.344
	5	-1.121	-1.140	-1.111	-1.124		15	-0.476	-0.503	-0.514	-0.497
	10	-1.550	-1.631	-1.524	-1.567		20	-0.575	-0.622	-0.609	-0.602
	15	-1.862	-1.910	-1.794	-1.854		30	-0.854	-0.840	-0.857	-0.850
78	0.5	-0.189	-0.188	-0.208	-0.195	45	2	-0.312	-0.340	-0.348	-0.333
	1	-0.363	-0.382	-0.336	-0.360		5	-0.773	-0.738	-0.780	-0.763
	2	-0.782	-0.873	-0.824	-0.826		10	-1.487	-1.563	-1.544	-1.531
	3	-1.180	-1.169	-1.146	-1.165		15	-2.238	-2.267	-2.207	-2.237
	5	-1.794	-1.921	-1.887	-1.866		20	-2.747	-2.787	-2.754	-2.762
	10	-3.225	-3.225	-3.310	-3.253		30	-3.466	-3.485	-3.438	-3.463
80	0.5	-0.208	-0.266	-0.262	-0.245						
	1	-0.641	-0.644	-0.657	-0.647						

	2	-1.479	-1.516	-1.497	-1.497
	3	-2.240	-2.244	-2.222	-2.235
	5	-3.936	-4.036	-4.049	-4.006
	10	-6.287	-6.064	-6.064	-6.133

GOx (Fig.4c)

		Ln(residual activity)			
temp	time (min)	#1	#2	#3	Mean
55	2	-0.420	-0.385	-0.427	-0.410
	5	-0.756	-0.838	-0.925	-0.837
	10	-1.345	-1.464	-1.526	-1.442
	15	-2.017	-2.059	-2.162	-2.077
	20	-2.338	-2.426	-2.267	-2.342
	30	-3.009	-3.322	-3.214	-3.173
60	2	-1.079	-1.144	-1.160	-1.127
	5	-2.828	-2.845	-2.848	-2.841
	10	-3.637	-3.652	-3.690	-3.659
	15	-4.448	-4.238	-4.553	-4.404
62	0.5	-0.505	-0.556	-0.530	-0.530
	1	-1.167	-1.244	-1.226	-1.212
	2	-2.298	-2.370	-2.426	-2.363
	5	-4.713	-4.499	-4.829	-4.671

(Fig.4d)

Control

	1/T	ln(k/T)
35	0.00325	-13.247
40	0.00319	-11.463
45	0.00314	-10.071

Mutant

	1/T	ln(k/T)
70	0.00291	-13.133
75	0.00287	-10.230
78	0.00285	-9.435
80	0.00283	-8.657

GOx

	1/T	ln(k/T)
55	0.00305	-10.422
60	0.00300	-9.602
62	0.00298	-8.751

Supplementary Data 3

Numerical Data for Fig.5

Control

[Glucose]	#1	#2	#3	Mean
0.000	0.054	0.039	0.023	0.039
0.100	0.072	0.072	0.170	0.105
0.997	0.168	0.170	0.176	0.172
2.985	0.248	0.293	0.332	0.291
4.966	0.309	0.399	0.478	0.395
9.882	0.432	0.574	0.692	0.566

Mutant

[Glucose]	#1	#2	#3	Mean
0.000	0.038	0.022	0.062	0.040
0.495	0.403	0.456	0.539	0.466
0.990	0.738	0.843	0.871	0.817
2.963	1.709	2.046	2.025	1.927
4.929	2.415	2.812	2.853	2.693
9.809	3.717	4.653	4.328	4.233

14.751	0.506	0.679	0.797	0.661	14.641	4.651	5.801	5.385	5.279
19.571	0.563	0.773	0.933	0.756	19.427	5.350	6.687	6.132	6.056

Supplementary Data 4

Numerical Data for Supplementary Figure 3

Control					Mutant				
[Glc]	#1	#2	#3	Mean	[Glc]	#1	#2	#3	Mean
100	113.876	133.265	106.612	117.918	100	232.204	291.342	273.079	265.541
50	101.041	91.483	73.186	88.570	50	241.398	251.585	226.551	239.845
20	75.644	89.298	71.439	78.794	20	185.614	185.614	166.978	179.402
10	61.990	59.532	47.626	56.383	10	120.264	131.197	113.058	121.506
5	39.597	58.167	46.533	48.099	5	76.904	76.532	78.706	77.381
2	14.473	26.216	20.973	20.554	2	33.420	33.669	38.266	35.118
1	1.092	6.281	5.025	4.133	1	15.157	17.394	23.481	18.677
0.5	7.100	0.546	0.437	2.694	0.5	6.212	6.088	13.480	8.593

Sequences of enzymes

Structural gene of enzyme was ligated to pTrc99A vector after Nco I/HIndIII (underlinded) digestion

<table>
<tr><td colspan="2">BcGDH αP205C/βD383C- γK155C/ βY349C</td></tr>
<tr><td>Nucleotide sequence</td><td>Amino acid sequence</td></tr>
<tr><td>CCATGGCACACAACGACAACACTCCCCACTCGCGTCG
CCACGGCGACGCAGCCGCATCAGGCATCACGCGGCGT
CAATGGTTGCAAGGCGCGCTGGCGCTGACCGCAGCGG
GCCTCACGGGTTCGCTGACATTGCGGGCGCTTGCAGA
CAACCCCGGCACTGCGCCGCTCGATACGTTCATGACG
CTTTCCGAATCGCTGACCGGCAAGAAAGGGCTCAGCC
GCGTGATCGGCGAGCGCCTGCTGCAGGCGCTGCAGAA
GGGCTCGTTCAAGACGGCCGACAGCCTGCCGCAGCTC
GCCGGCGCGCTCGCGTCCGGTTCGCTGACGCCTGAAC
AGGAATCGCTCGCACTGACGATCCTCGAGGCCTGGTA
TCTCGGCATCGTCGACAACGTCGTGATTACGTACGAG
GAAGCATTAATGTTCGGCGTCGTGTCCGATACGCTCG
TGATCCGTTCGTATTGCCCCAACTGTCCCGGCTTCTG
GGCCGACAAACCGATCGAGAGGCAAGCCTGATGGCCG
ATACCGATACGCAAAAGGCCGACGTCGTCGTCGTTGG
ATCGGGTGTCGCGGGCGCGATCGTCGCGCATCAGCTC
GCGATGGCGGGCAAGGCGGTGATCCTGCTCGAAGCGG
GCCCGCGCATGCCGCGCTGGGAAATCGTCGAGCGCTT
CCGCAATCAGCCCGACAAGATGGACTTCATGGCGCCG
TACCCGTCGAGCCCCTGGGCGCCGCATCCCGAGTACG
GCCCGCCGAACGACTACCTGATCCTGAAGGGTGAACA
CAAGTTCAACTCTCAGTATATTCGTGCAGTAGGTGGT
ACTACTTGGCACTGGGCTGCATCTGCATGGCGTTTCA
TTCCGAACGACTTCAAGATGAAGAGCGTGTACGGCGT
CGGCCGCGACTGGCCGATCCAGTACGACGATCTCGAG
CCGTACTATCAGCGCGCGGAGGAAGAGCTCGGCGTGT
GGGGCCCGGGCCCCGAGGAAGATCTGTACTCGCCGCG
CAAGCAGCCGTATCCGATGCCGCCGCTGCCGTTGTCG
TTCAACGAGCAGACCATCAAGACGGCGCTGAACAACT
ACGATCCGAAGTTCCATGTCGTGACCGAGCCAGTTGC
ACGTAACTCTCGTTGCTACGACGGTCGTCCAACTTGT
TGCGGCAACAACAACTGCATGCCGATCTGCCCGATCG
GCGCGATGTACAACGGCATCGTGCACGTCGAGAAGGC
CGAACGCGCCGGCGCGAAGCTGATCGAGAACGCGGTC
GTCTACAAGCTCGAGACGGGCCCGGACAAGCGCATCG
TCGCGGCGCTCTACAAGGACAAGACGGGCGCCGAGCA
TCGCGTCGAAGGCAAGTATTTCGTGCTCGCCGCGAAC
GGCATCGAGACGCCGAAGATCCTGCTGATGTCCGCGA
ACCGCGATTTCCCGAACGGTGTCGCGAACAGCTCGGA
CATGGTTGGTCGTAACCTGATGGATCATCCAGGTACT
GGTGTACAGTTCTATGCGAGCGAGAAGCTGTGGCCAG
GTCGTGGTCCACAGGAGATGACGTCGCTGATCGGTTT
CCGCGATGGTCCATTCCGTGCAACTGAAGCAGCTAAG
AAGATTCACCTGTACAACCTGTCTCGTATCGACCAGG
AGACGCAGAAGATCTTCAAGGCCGGCAAGCTGATGAA
GCCCGACGAGCTGGATGCTCAGATTCGTGATCGTTCT
GCACGTTATGTACAGTTCGACTGCTTCCACGAAATCC
TGCCGCAACCCGAGAACCGCATCGTGCCGAGCAAGAC
GGCGACCGATGCGATCGGCATTCCGCGCCCCGAGATC
ACGTATGCGATCGACGACTACGTGAAGCGTGGTGCAG</td><td>MAHNDNTPHSRRHGDAAASGITRRQWLQGALALTAAG
LTGSLTLRALADNPGTAPLDTFMTLSESLTGKKGLSR
VIGERLLQALQKGSFKTADSLPQLAGALASGSLTPEQ
ESLALTILEAWYLGIVDNVVITYEEALMFGVVSDTLV
IRSYCPNCPGFWADKPIERQA*

MADTDTQKADVVVVGSGVAGAIVAHQLAMAGKAVILL
EAGPRMPRWEIVERFRNQPDKMDFMAPYPSSPWAPHP
EYGPPNDYLILKGEHKFNSQYIRAVGGTTWHWAASAW
RFIPNDFKMKSVYGVGRDWPIQYDDLEPYYQRAEEEL
GVWGPGPEEDLYSPRKQPYPMPPLPLSFNEQTIKTAL
NNYDPKFHVVTEPVARNSRCYDGRPTCCGNNNCMPIC
PIGAMYNGIVHVEKAERAGAKLIENAVVYKLETGPDK
RIVAALYKDKTGAEHRVEGKYFVLAANGIETPKILLM
SANRDFPNGVANSSDMVGRNLMDHPGTGVQFYASEKL
WPGRGPQEMTSLIGFRDGPFRATEAAKKIHLYNLSRI
DQETQKIFKAGKLMKPDELDAQIRDRSARYVQFDCFH
EILPQPENRIVPSKTATDAIGIPRPEITYAIDDYVKR
GAAHTREVYATAAKVLGGTDVVFNDEFYPNNHITGST
IMGADARDSVVDKDCRTFDHPNLFISSSATMPTVGTV
NVTLTIAALALRMSDTLKKEV*

MRKSTLTFLIAGCLALPGFARAADAADPALVKRGEYL
ATAGDCMACHTVKGGKPYAGGLGMPVPMLGKIYTSNI
TPDPDTGIGKWTFEDFERAVRHGVSKNGDNLYPAMPY
VSYAKITDDDVRALYAYFMHGVEPVKQAPPKNEIPAL
LSMRWPLKIWNWLFLKDGPYQPKPSQSAEWNRGAYLV
QGLAHCSTCHTPRGIAMQEKSLDETGGSFLAGSVLAG
WDGYNITSDPNAGIGSWTQQQLVQYLRTGSVPGVAQA
AGPMAEAVEHSFSKMTEADIGAIATYVRTVPAVADSN
AKQPRSSWGKPAEDGLKLRGVALASSGIDPARLYLGN
CATCHQMQGKGTPDGCYPSLFHNSTVGASNPSNLVQV
ILNGVQRKIGSECIGMPAFRYDLNDAQIAALTNYVTA
QFGNPAAKVTEQDVAKLR*</td></tr>
</table>

CTCATACTCGTGAAGTATACGCTACCGCAGCTAAAGT<br>ACTGGGTGGTACTGACGTTGTATTCAACGACGAATTC<br>TACCCGAACAATCACATCACGGGCTCGACGATCATGG<br>GCGCCGATGCGCGCGACTCCGTCGTCGACAAGGACTG<br>CCGCACGTTCGACCATCCGAACCTGTTCATTTCGAGC<br>AGCGCGACGATGCCGACCGTCGGTACCGTAAACGTGA<br>CGCTGACGATCGCCGCGCTCGCGCTGCGGATGTCGGA<br>CACGCTGAAGAAGGAAGTCTGACCATGCGGAAATCTA<br>CTCTCACTTTCCTCATCGCCGGCTGCCTCGCGTTGCC<br>GGGCTTCGCGCGCGCGGCCGATGCGGCCGATCCGGCG<br>CTGGTCAAGCGCGGCGAATACCTCGCGACCGCCGGCG<br>ACTGCATGGCCTGCCACACCGTGAAGGGCGGCAAGCC<br>GTACGCGGGCGGCCTTGGCATGCCGGTACCGATGCTC<br>GGCAAGATCTACACGAGCAACATCACGCCCGATCCCG<br>ATACGGGCATCGGCAAATGGACGTTCGAGGACTTCGA<br>GCGCGCGGTGCGGCACGGCGTGTCGAAGAACGGCGAC<br>AACCTGTATCCGGCGATGCCGTACGTGTCGTACGCGA<br>AGATCACGGACGACGACGTACGCGCGCTGTACGCCTA<br>CTTCATGCACGGCGTCGAGCCGGTCAAGCAGGCGCCG<br>CCGAAGAACGAGATTCCCGCGCTGCTCAGCATGCGCT<br>GGCCGCTGAAGATCTGGAACTGGCTGTTCCTGAAGGA<br>CGGCCCGTACCAGCCGAAGCCGTCGCAGAGCGCCGAA<br>TGGAATCGCGGCGCGTATCTGGTGCAGGGTCTCGCGC<br>ACTGCAGCACGTGCCACACGCCGCGCGGCATCGCGAT<br>GCAGGAGAAGTCGCTCGACGAAACCGGCGGCAGCTTC<br>CTCGCGGGGTCGGTGCTCGCCGGCTGGGACGGCTACA<br>ACATCACGTCGGACCCGAATGCGGGGATCGGCAGCTG<br>GACGCAGCAGCAGCTCGTGCAGTATTTGCGCACCGGC<br>AGCGTGCCGGGCGTCGCGCAGGCGGCCGGGCCGATGG<br>CCGAGGCGGTCGAGCACAGCTTCTCGAAGATGACCGA<br>AGCGGACATCGGTGCGATCGCCACGTACGTCCGCACG<br>GTGCCGGCCGTTGCCGACAGCAACGCGAAGCAGCCGC<br>GGTCGTCGTGGGGCAAGCCGGCCGAGGACGGGCTGAA<br>GCTGCGCGGTGTCGCGCTCGCGTCGTCGGGCATCGAT<br>CCGGCGCGGCTGTATCTCGGCAACTGCGCGACGTGCC<br>ACCAGATGCAGGGCAAGGGCACGCCGGACGGCTGTTA<br>CCCGTCGCTGTTCCACAACTCCACCGTCGGCGCGTCG<br>AATCCGTCGAACCTCGTGCAGGTGATCCTGAACGGCG<br>TGCAGCGCAAGATCGGCAGCGAGTGTATCGGGATGCC<br>CGCTTTCCGCTACGATCTGAACGACGCGCAGATCGCC<br>GCGCTGACGAACTACGTGACCGCGCAGTTCGGCAATC<br>CGGCGGCGAAGGTGACGGAGCAGGACGTCGCGAAGCT<br>GCGCTGAAAGCTT	
BcGDH control for enzyme assay	
Nucleotide sequence	Amino acid sequence
CCATGGCACACAACGACAACACTCCCCACTCGCGTCG<br>CCACGGCGACGCAGCCGCATCAGGCATCACGCGGCGT<br>CAATGGTTGCAAGGCGCGCTGGCGCTGACCGCAGCGG<br>GCCTCACGGGTTCGCTGACATTGCGGGCGCTTGCAGA<br>CAACCCCGGCACTGCGCCGCTCGATACGTTCATGACG<br>CTTTCCGAATCGCTGACCGGCAAGAAAGGGCTCAGCC<br>GCGTGATCGGCGAGCGCCTGCTGCAGGCGCTGCAGAA<br>GGGCTCGTTCAAGACGGCCGACAGCCTGCCGCAGCTC<br>GCCGGCGCGCTCGCGTCCGGTTCGCTGACGCCTGAAC<br>AGGAATCGCTCGCACTGACGATCCTCGAGGCCTGGTA<br>TCTCGGCATCGTCGACAACGTCGTGATTACGTACGAG	MAHNDNTPHSRRHGDAAASGITRRQWLQGALALTAAG<br>LTGSLTLRALADNPGTAPLDTFMTLSESLTGKKGLSR<br>VIGERLLQALQKGSFKTADSLPQLAGALASGSLTPEQ<br>ESLALTILEAWYLGIVDNVVITYEEALMFGVVSDTLV<br>IRSYCPNKPGFWADKPIERQA*<br><br>MADTDTQKADVVVVGSGVAGAIVAHQLAMAGKAVILL<br>EAGPRMPRWEIVERFRNQPDKMDFMAPYPSSPWAPHP<br>EYGPPNDYLILKGEHKFNSQYIRAVGGTTWHWAASAW<br>RFIPNDFKMKSVYGVGRDWPIQYDDLEPYYQRAEEEL<br>GVWGPGPEEDLYSPRKQPYPMPPLPLSFNEQTIKTAL

GAAGCATTAATGTTCGGCGTCGTGTCCGATACGCTCG	NNYDPKFHVVTEPVARNSRPYDGRPTCCGNNNCMPIC
TGATCCGTTCGTATTGCCCCAACAAACCCGGCTTCTG	PIGAMYNGIVHVEKAERAGAKLIENAVVYKLETGPDK
GGCCGACAAACCGATCGAGAGGCAAGCCTGATGGCCG	RIVAALYKDKTGAEHRVEGKYFVLAANGIETPKILLM
ATACCGATACGCAAAAGGCCGACGTCGTCGTCGTTGG	SANRDFPNGVANSSDMVGRNLMDHPGTGVQFYASEKL
ATCGGGTGTCGCGGGCGCGATCGTCGCGCATCAGCTC	WPGRGPQEMTSLIGFRDGPFRATEAAKKIHLYNLSRI
GCGATGGCGGGCAAGGCGGTGATCCTGCTCGAAGCGG	DQETQKIFKAGKLMKPDELDAQIRDRSARYVQFDCFH
GCCCGCGCATGCCGCGCTGGGAAATCGTCGAGCGCTT	EILPQPENRIVPSKTATDAIGIPRPEITYAIDDYVKR
CCGCAATCAGCCCGACAAGATGGACTTCATGGCGCCG	GAAHTREVYATAAKVLGGTDVVFNDEFYPNNHITGST
TACCCGTCGAGCCCCTGGGCGCCGCATCCCGAGTACG	IMGADARDSVVDKDCRTFDHPNLFISSSATMPTVGTV
GCCCGCCGAACGACTACCTGATCCTGAAGGGTGAACA	NVTLTIAALALRMSDTLKKEV*
CAAGTTCAACTCTCAGTATATTCGTGCAGTAGGTGGT	
ACTACTTGGCACTGGGCTGCATCTGCATGGCGTTTCA	MRKSTLTFLIAGCLALPGFARAADAADPALVKRGEYL
TTCCGAACGACTTCAAGATGAAGAGCGTGTACGGCGT	ATAGDCMACHTVKGGKPYAGGLGMPVPMLGKIYTSNI
CGGCCGCGACTGGCCGATCCAGTACGACGATCTCGAG	TPDPDTGIGKWTFEDFERAVRHGVSKNGDNLYPAMPY
CCGTACTATCAGCGCGCGGAGGAAGAGCTCGGCGTGT	VSYAKITDDDVRALYAYFMHGVEPVKQAPPKNEIPAL
GGGGCCCGGGCCCCGAGGAAGATCTGTACTCGCCGCG	LSMRWPLKIWNWLFLKDGPYQPKPSQSAEWNRGAYLV
CAAGCAGCCGTATCCGATGCCGCCGCTGCCGTTGTCG	QGLAHCSTCHTPRGIAMQEKSLDETGGSFLAGSVLAG
TTCAACGAGCAGACCATCAAGACGGCGCTGAACAACT	WDGYNITSDPNAGIGSWTQQQLVQYLRTGSVPGVAQA
ACGATCCGAAGTTCCATGTCGTGACCGAGCCAGTTGC	AGPMAEAVEHSFSKMTEADIGAIATYVRTVPAVADSN
ACGTAACTCTCGTCCATACGACGGTCGTCCAACTTGT	AKQPRSSWGKPAEDGLKLRGVALASSGIDPARLYLGN
TGCGGCAACAACAACTGCATGCCGATCTGCCCGATCG	CATCHQMQGKGTPDGYYPSLFHNSTVGASNPSNLVQV
GCGCGATGTACAACGGCATCGTGCACGTCGAGAAGGC	ILNGVQRKIGSEDIGMPAFRYDLNDAQIAALTNYVTA
CGAACGCGCCGGCGCGAAGCTGATCGAGAACGCGGTC	QFGNPAAKVTEQDVAKLR*
GTCTACAAGCTCGAGACGGGCCCGGACAAGCGCATCG	
TCGCGGCGCTCTACAAGGACAAGACGGGCGCCGAGCA	
TCGCGTCGAAGGCAAGTATTTCGTGCTCGCCGCGAAC	
GGCATCGAGACGCCGAAGATCCTGCTGATGTCCGCGA	
ACCGCGATTTCCCGAACGGTGTCGCGAACAGCTCGGA	
CATGGTTGGTCGTAACCTGATGGATCATCCAGGTACT	
GGTGTACAGTTCTATGCGAGCGAGAAGCTGTGGCCAG	
GTCGTGGTCCACAGGAGATGACGTCGCTGATCGGTTT	
CCGCGATGGTCCATTCCGTGCAACTGAAGCAGCTAAG	
AAGATTCACCTGTACAACCTGTCTCGTATCGACCAGG	
AGACGCAGAAGATCTTCAAGGCCGGCAAGCTGATGAA	
GCCCGACGAGCTGGATGCTCAGATTCGTGATCGTTCT	
GCACGTTATGTACAGTTCGACTGCTTCCACGAAATCC	
TGCCGCAACCCGAGAACCGCATCGTGCCGAGCAAGAC	
GGCGACCGATGCGATCGGCATTCCGCGCCCCGAGATC	
ACGTATGCGATCGACGACTACGTGAAGCGTGGTGCAG	
CTCATACTCGTGAAGTATACGCTACCGCAGCTAAAGT	
ACTGGGTGGTACTGACGTTGTATTCAACGACGAATTC	
TACCCGAACAATCACATCACGGGCTCGACGATCATGG	
GCGCCGATGCGCGCGACTCCGTCGTCGACAAGGACTG	
CCGCACGTTCGACCATCCGAACCTGTTCATTTCGAGC	
AGCGCGACGATGCCGACCGTCGGTACCGTAAACGTGA	
CGCTGACGATCGCCGCGCTCGCGCTGCGGATGTCGGA	
CACGCTGAAGAAGGAAGTCTGACCATGCGGAAATCTA	
CTCTCACTTTCCTCATCGCCGGCTGCCTCGCGTTGCC	
GGGCTTCGCGCGCGCGGCCGATGCGGCCGATCCGGCG	
CTGGTCAAGCGCGGCGAATACCTCGCGACCGCCGGCG	
ACTGCATGGCCTGCCACACCGTGAAGGGCGGCAAGCC	
GTACGCGGGCGGCCTTGGCATGCCGGTACCGATGCTC	
GGCAAGATCTACACGAGCAACATCACGCCCGATCCCG	
ATACGGGCATCGGCAAATGGACGTTCGAGGACTTCGA	
GCGCGCGGTGCGGCACGGCGTGTCGAAGAACGGCGAC	
AACCTGTATCCGGCGATGCCGTACGTGTCGTACGCGA	
AGATCACGGACGACGACGTACGCGCGCTGTACGCCTA	

CTTCATGCACGGCGTCGAGCCGGTCAAGCAGGCGCCG	
CCGAAGAACGAGATTCCCGCGCTGCTCAGCATGCGCT	
GGCCGCTGAAGATCTGGAACTGGCTGTTCCTGAAGGA	
CGGCCCGTACCAGCCGAAGCCGTCGCAGAGCGCCGAA	
TGGAATCGCGGCGCGTATCTGGTGCAGGGTCTCGCGC	
ACTGCAGCACGTGCCACACGCCGCGCGGCATCGCGAT	
GCAGGAGAAGTCGCTCGACGAAACCGGCGGCAGCTTC	
CTCGCGGGGTCGGTGCTCGCCGGCTGGGACGGCTACA	
ACATCACGTCGGACCCGAATGCGGGGATCGGCAGCTG	
GACGCAGCAGCAGCTCGTGCAGTATTTGCGCACCGGC	
AGCGTGCCGGGCGTCGCGCAGGCGGCCGGGCCGATGG	
CCGAGGCGGTCGAGCACAGCTTCTCGAAGATGACCGA	
AGCGGACATCGGTGCGATCGCCACGTACGTCCGCACG	
GTGCCGGCCGTTGCCGACAGCAACGCGAAGCAGCCGC	
GGTCGTCGTGGGGCAAGCCGGCCGAGGACGGGCTGAA	
GCTGCGCGGTGTCGCGCTCGCGTCGTCGGGCATCGAT	
CCGGCGCGGCTGTATCTCGGCAACTGCGCGACGTGCC	
ACCAGATGCAGGGCAAGGGCACGCCGGACGGCTATTA	
CCCGTCGCTGTTCCACAACTCCACCGTCGGCGCGTCG	
AATCCGTCGAACCTCGTGCAGGTGATCCTGAACGGCG	
TGCAGCGCAAGATCGGCAGCGAGGATATCGGGATGCC	
CGCTTTCCGCTACGATCTGAACGACGCGCAGATCGCC	
GCGCTGACGAACTACGTGACCGCGCAGTTCGGCAATC	
CGGCGGCGAAGGTGACGGAGCAGGACGTCGCGAAGCT	
GCGCTGAAAGCTT	
BcGDH for crystallization (His-tagged)	
Nucleotide sequence	Amino acid sequence
CCATGCACAACGACAACACTCCCCACTCGCGTCGCCA	MHNDNTPHSRRHGDAAASGITRRQWLQGALALTAAGL
CGGCGACGCAGCCGCATCAGGCATCACGCGGCGTCAA	TGSLTLRALADNPGTAPLDTFMTLSESLTGKKGLSRV
TGGTTGCAAGGCGCGCTGGCGCTGACCGCAGCGGGCC	IGERLLQALQKGSFKTADSLPQLAGALASGSLTPEQE
TCACGGGTTCGCTGACATTGCGGGCGCTTGCAGACAA	SLALTILEAWYLGIVDNVVITYEEALMFGVVSDTLVI
CCCCGGCACTGCGCCGCTCGATACGTTCATGACGCTT	RSYCPNKPGFWADKPIERQA*
TCCGAATCGCTGACCGGCAAGAAAGGGCTCAGCCGCG	
TGATCGGCGAGCGCCTGCTGCAGGCGCTGCAGAAGGG	MADTDTQKADVVVVGSGVAGAIVAHQLAMAGKAVILL
CTCGTTCAAGACGGCCGACAGCCTGCCGCAGCTCGCC	EAGPRMPRWEIVERFRNQPDKMDFMAPYPSSPWAPHP
GGCGCGCTCGCGTCCGGTTCGCTGACGCCTGAACAGG	EYGPPNDYLILKGEHKFNSQYIRAVGGTTWHWAASAW
AATCGCTCGCACTGACGATCCTCGAGGCCTGGTATCT	RFIPNDFKMKSVYGVGRDWPIQYDDLEPYYQRAEEEL
CGGCATCGTCGACAACGTCGTGATTACGTACGAGGAA	GVWGPGPEEDLYSPRKQPYPMPPLPLSFNEQTIKTAL
GCATTAATGTTCGGCGTCGTGTCCGATACGCTCGTGA	NNYDPKFHVVTEPVARNSRPYDGRPTCCGNNNCMPIC
TCCGTTCGTATTGCCCCAACAAACCCGGCTTCTGGGC	PIGAMYNGIVHVEKAERAGAKLIENAVVYKLETGPDK
CGACAAACCGATCGAGAGGCAAGCCTGATGGCCGATA	RIVAALYKDKTGAEHRVEGKYFVLAANGIETPKILLM
CCGATACGCAAAAGGCCGACGTCGTCGTCGTTGGATC	SANRDFPNGVANSSDMVGRNLMDHPGTGVSFYASEKL
GGGTGTCGCGGGCGCGATCGTCGCGCATCAGCTCGCG	WPGRGPQEMTSLIGFRDGPFRATEAAKKIHLSNLSRI
ATGGCGGGCAAGGCGGTGATCCTGCTCGAAGCGGGCC	DQETQKIFKAGKLMKPDELDAQIRDRSARYVQFDCFH
CGCGCATGCCGCGCTGGGAAATCGTCGAGCGCTTCCG	EILPQPENRIVPSKTATDAIGIPRPEITYAIDDYVKR
CAATCAGCCCGACAAGATGGACTTCATGGCGCCGTAC	GAAHTREVYATAAKVLGGTDVVFNDEFAPNNHITGST
CCGTCGAGCCCCTGGGCGCCGCATCCCGAGTACGGCC	IMGADARDSVVDKDCRTFDHPNLFISSSATMPTVGTV
CGCCGAACGACTACCTGATCCTGAAGGGCGAGCACAA	NVTLTIAALALRMSDTLKKEV*
GTTCAACTCGCAGTACATCCGCGCGGTGGGCGGCACG	
ACGTGGCACTGGGCCGCGTCGGCGTGGCGCTTCATTC	MRKSTLTFLIAGCLALPGFARAADAADPALVKRGEYL
CGAACGACTTCAAGATGAAGAGCGTGTACGGCGTCGG	ATAGDCMACHTVKGGKPYAGGLGMPVPMLGKIYTSNI
CCGCGACTGGCCGATCCAGTACGACGATCTCGAGCCG	TPDPDTGIGKWTFEDFERAVRHGVSKNGDNLYPAMPY
TACTATCAGCGCGCGGAGGAAGAGCTCGGCGTGTGGG	VSYAKITDDDVRALYAYFMHGVEPVKQAPPKNEIPAL
GCCCGGGCCCCGAGGAAGATCTGTACTCGCCGCGCAA	LSMRWPLKIWNWLFLKDGPYQPKPSQSAEWNRGAYLV
GCAGCCGTATCCGATGCCGCCGCTGCCGTTGTCGTTC	QGLAHCSTCHTPRGIAMQEKSLDETGGSFLAGSVLAG

AACGAGCAGACCATCAAGACGGCGCTGAACAACTACG	WDGYNITSDPNAGIGSWTQQQLVQYLRTGSVPGVAQA
ATCCGAAGTTCCATGTCGTGACCGAGCCGGTCGCGCG	AGPMAEAVEHSFSKMTEADIGAIATYVRTVPAVADSN
CAACAGCCGCCCGTACGACGGCCGCCCGACTTGTTGC	AKQPRSSWGKPAEDGLKLRGVALASSGIDPARLYLGN
GGCAACAACAACTGCATGCCGATCTGCCCGATCGGCG	CATCHQMQGKGTPDGYYPSLFHNSTVGASNPSNLVQV
CGATGTACAACGGCATCGTGCACGTCGAGAAGGCCGA	ILNGVQRKIGSEDIGMPAFRYDLNDAQIAALTNYVTA
ACGCGCCGGCGCGAAGCTGATCGAGAACGCGGTCGTC	QFGNPAAKVTEQDVAKLRAGGKQVPLAKLASPGVMLL
TACAAGCTCGAGACGGGCCCGGACAAGCGCATCGTCG	LGTGGILGAILVVAGLWWLISRRKKRSAHHHHHH*
CGGCGCTCTACAAGGACAAGACGGGCGCCGAGCATCG	
CGTCGAAGGCAAGTATTTCGTGCTCGCCGCGAACGGC	
ATCGAGACGCCGAAGATCCTGCTGATGTCCGCGAACC	
GCGATTTCCCGAACGGTGTCGCGAACAGCTCGGACAT	
GGTCGGCCGCAACCTGATGGACCATCCGGGCACCGGC	
GTGTCGTTCTATGCGAGCGAGAAGCTGTGGCCGGGCC	
GCGGCCCGCAGGAGATGACGTCGCTGATCGGTTTCCG	
CGACGGTCCGTTCCGCGCGACCGAAGCGGCGAAGAAG	
ATCCACCTGTCGAACCTGTCGCGCATCGACCAGGAGA	
CGCAGAAGATCTTCAAGGCCGGCAAGCTGATGAAGCC	
CGACGAGCTCGACGCGCAGATCCGCGACCGTTCCGCA	
CGCTACGTGCAGTTCGACTGCTTCCACGAAATCCTGC	
CGCAACCCGAGAACCGCATCGTGCCGAGCAAGACGGC	
GACCGATGCGATCGGCATTCCGCGCCCCGAGATCACG	
TATGCGATCGACGACTACGTGAAGCGCGGCGCCGCGC	
ATACGCGCGAGGTCTACGCGACCGCCGCGAAGGTGCT	
CGGCGGCACGGACGTCGTGTTCAACGACGAATTCGCG	
CCGAACAATCACATCACGGGCTCGACGATCATGGGCG	
CCGATGCGCGCGACTCCGTCGTCGACAAGGACTGCCG	
CACGTTCGACCATCCGAACCTGTTCATTTCGAGCAGC	
GCGACGATGCCGACCGTCGGTACCGTAAACGTGACGC	
TGACGATCGCCGCGCTCGCGCTGCGGATGTCGGACAC	
GCTGAAGAAGGAAGTCTGACCATGCGGAAATCTACTC	
TCACTTTCCTCATCGCCGGCTGCCTCGCGTTGCCGGG	
CTTCGCGCGCGCGGCCGATGCGGCCGATCCGGCGCTG	
GTCAAGCGCGGCGAATACCTCGCGACCGCCGGCGACT	
GCATGGCCTGCCACACCGTGAAGGGCGGCAAGCCGTA	
CGCGGGCGGCCTTGGCATGCCGGTACCGATGCTCGGC	
AAGATCTACACGAGCAACATCACGCCCGATCCCGATA	
CGGGCATCGGCAAATGGACGTTCGAGGACTTCGAGCG	
CGCGGTGCGGCACGGCGTGTCGAAGAACGGCGACAAC	
CTGTATCCGGCGATGCCGTACGTGTCGTACGCGAAGA	
TCACGGACGACGACGTACGCGCGCTGTACGCCTACTT	
CATGCACGGCGTCGAGCCGGTCAAGCAGGCGCCGCCG	
AAGAACGAGATTCCCGCGCTGCTCAGCATGCGCTGGC	
CGCTGAAGATCTGGAACTGGCTGTTCCTGAAGGACGG	
CCCGTACCAGCCGAAGCCGTCGCAGAGCGCCGAATGG	
AATCGCGGCGCGTATCTGGTGCAGGGTCTCGCGCACT	
GCAGCACGTGCCACACGCCGCGCGGCATCGCGATGCA	
GGAGAAGTCGCTCGACGAAACCGGCGGCAGCTTCCTC	
GCGGGGTCGGTGCTCGCCGGCTGGGACGGCTACAACA	
TCACGTCGGACCCGAATGCGGGGATCGGCAGCTGGAC	
GCAGCAGCAGCTCGTGCAGTATTTGCGCACCGGCAGC	
GTGCCGGGCGTCGCGCAGGCGGCCGGGCCGATGGCCG	
AGGCGGTCGAGCACAGCTTCTCGAAGATGACCGAAGC	
GGACATCGGTGCGATCGCCACGTACGTCCGCACGGTG	
CCGGCCGTTGCCGACAGCAACGCGAAGCAGCCGCGGT	
CGTCGTGGGGCAAGCCGGCCGAGGACGGGCTGAAGCT	
GCGCGGTGTCGCGCTCGCGTCGTCGGGCATCGATCCG	
GCGCGGCTGTATCTCGGCAACTGCGCGACGTGCCACC	

AGATGCAGGGCAAGGGCACGCCGGACGGCTATTACCC<br>GTCGCTGTTCCACAACTCCACCGTCGGCGCGTCGAAT<br>CCGTCGAACCTCGTGCAGGTGATCCTGAACGGCGTGC<br>AGCGCAAGATCGGCAGCGAGGATATCGGGATGCCCGC<br>TTTCCGCTACGATCTGAACGACGCGCAGATCGCCGCG<br>CTGACGAACTACGTGACCGCGCAGTTCGGCAATCCGG<br>CGGCGAAGGTGACGGAGCAGGACGTCGCGAAGCTGCG<br>CGCCGGTGGGAAACAGGTCCCCTTAGCTAAGTTGGCT<br>AGTCCAGGTGTCATGTTACTTCTGGGAACTGGGGGCA<br>TTCTGGGAGCTATTCTTGTTGTAGCGGGACTGTGGTG<br>GCTGATTTCTCGTCGTAAGAAGCGCAGCGCTCACCAC<br>CACCACCACCACTGAAGCTT	
